# Novel Nanocomposites and Biopolymer-Based Nanocomposites for Hexavalent Chromium Removal from Aqueous Media

**DOI:** 10.3390/polym16243469

**Published:** 2024-12-12

**Authors:** Adina-Elena Segneanu, Ionela Amalia Bradu, Mihaela Simona Calinescu (Bocanici), Gabriela Vlase, Titus Vlase, Daniel-Dumitru Herea, Gabriela Buema, Maria Mihailescu, Ioan Grozescu

**Affiliations:** 1 Institute for Advanced Environmental Research, West University of Timisoara (ICAM-WUT), 4 Oituz St., 300086 Timișoara, Romania; ionela.bradu@e-uvt.ro (I.A.B.); gabriela.vlase@e-uvt.ro (G.V.); titus.vlase@e-uvt.ro (T.V.); 2 Faculty of Chemistry, Biology, Geography, West University of Timisoara (ICAM-WUT), Pestalozzi St. 16, 300115 Timișoara, Romania; mihaela.calinescu88@e-uvt.ro; 3 Research Center for Thermal Analyzes in Environmental Problems, Problems, West University of Timisoara (ICAM-WUT), Pestalozzi St. 16, 300115 Timișoara, Romania; 4 National Institute of Research and Development for Technical Physics, 47 Mangeron Blvd, 700050 Iasi, Romania; dherea@phys-iasi.ro (D.-D.H.); gbuema@phys-iasi.ro (G.B.); 5 Department of Applied Chemistry and Engineering of Inorganic Compounds and the Environment, University Politehnica Timisoara, 2 Piata Victoriei, 300006 Timișoara, Romania; mihailescumia@gmail.com (M.M.); ioangrozescu@gmail.com (I.G.); 6 Research Institute for Renewable Energy, 138 Gavril Musicescu St., 300501 Timișoara, Romania

**Keywords:** biopolymer-based composite, water remediation, adsorbent, wastes, heavy metal pollution

## Abstract

Designing new engineered materials derived from waste is essential for effective environmental remediation and reducing anthropogenic pollution in our economy. This study introduces an innovative method for remediating metal-contaminated water, using two distinct waste types: one biowaste (eggshell) and one industrial waste (fly ash). We synthesized three novel, cost-effective nanoadsorbent types, including two new tertiary composites and two biopolymer-based composites (specifically k-carrageenan and chitosan), which targeted chromium removal from aqueous solutions. SEM analysis reveals that in the first composite, EMZ, zeolite, and magnetite nanoparticles are successfully integrated into the porous structure of the eggshell. In the second composite (FMZ), fly ash and magnetite particles are similarly loaded within the zeolite pores. Each biopolymer-based composite is derived by incorporating the corresponding tertiary composite (FMZ or EMZ) into the biopolymer framework. Structural modifications of the eggshell, zeolite, chitosan, and k-carrageenan resulted in notable increases in specific surface area, as confirmed by BET analysis. These enhancements significantly improve chromium adsorption efficiency for each adsorbent type developed. The adsorption performances achieved are as follows: EMZ (89.76%), FMZ (84.83%), EMZCa (96.64%), FMZCa (94.87%), EMZC (99.64%), and FMZC (97.67%). The findings indicate that chromium adsorption across all adsorbent types occurs via a multimolecular layer mechanism, which is characterized as spontaneous and endothermic. Desorption studies further demonstrate the high reusability of these nanomaterials. Overall, this research underscores the potential of utilizing waste materials for new performant engineered low-cost composites and biocomposites for environmental bioremediation applications.

## 1. Introduction

The coexistence of chromium in two oxidation states, Cr(III) and Cr(VI), poses a significant challenge in managing water pollution. While Cr(III) is stable and naturally abundant, Cr(VI) is a highly toxic oxidizing agent prevalent in industrial settings due to human activities like leather tanning, glass manufacturing, ceramics, textiles, wood preservation, and the production of certain adhesives, which pose health risks to humans and ecosystems [1,2,3,4,5,6].

Chromium (VI) is recognized as one of the most hazardous metallic pollutants associated with significant health risks, which include genetic mutations and carcinogenic effects [7,8]. This pollutant is regulated by stringent standards established by the World Health Organization (WHO), which limits its concentration in drinking water to 0.05 mg/L, while European Union legislation restricts it to a maximum of 0.1 mg/L. Exposure to chromium (VI) is correlated with numerous health issues, such as genetic alterations, cancer, skin irritation, ulcers, and oxidative stress, which lead to considerable biological damage [7,8].

The highly mobile and soluble nature of chromium (VI) in aquatic ecosystems complicates its removal through conventional water treatment methods [7,8]. Current methods for removing hexavalent chromium (Cr(VI)) from polluted water sources include chemical precipitation, ion exchange, and membrane filtration. However, each of these techniques presents certain limitations regarding efficiency, cost-effectiveness, and environmental impact [1,4,5,6,9]:

Chemical precipitation is relatively straightforward but generates sludge, which requires additional treatment and disposal [1,4,5,6,9].

Ion Exchange can be effective but is often resource-intensive, which leads to increased operational costs and material consumption [1,4,5,6,9].

Coagulation and reverse osmosis are reliable but typically involve high capital and operational expenses, which make them less accessible for widespread use [1,4,5,6,9].

Adsorption offers versatility but we must carefully balance efficacy with economic considerations as traditional adsorbents can be costly [1,4,5,6,9]. Magnetic nanoparticles, zeolite, fly ash, and hybrid materials demonstrate good potential for immobilizing several pollutant categories from wastewater [1,4,5,6,9,10,11,12,13,14,15].

Given these challenges, there is a pressing need for innovative approaches that can enhance the efficiency and sustainability of chromium extraction from aquatic environments. Ongoing research is focused on developing new materials and methods that can overcome these limitations while minimizing environmental impact and operational costs.

Recent studies have focused on eco-friendly biocomposites that combine biopolymers or plant-derived materials with inorganic or organic components [1,16]. These biocomposites offer ecological benefits, enhanced adsorption efficiency, and cost-effectiveness [1,16]. Several studies have reported the development of novel adsorbents from natural products, such as agricultural waste (e.g., fruit husks, eggshells), natural proteins (e.g., gelatin, whey proteins), and natural polysaccharides (e.g., agarose, pectin, carrageenan) [17,18,19]. While natural adsorbents are easily accessible and cost-effective, engineered materials provide superior adsorption capacities due to their increased surface area and optimized pore structures [20,21,22]. These advanced materials also exhibit improved selectivity and stability, ensuring more efficient and reliable performance across various applications. Furthermore, combining microbial biomass (e.g., bacteria and fungi) with magnetic materials enhances after-adsorption separation, increasing both the specific surface area and adsorption capacity [23]. Additionally, functionalizing materials such as chitosan, cellulose, and alginate with metal nanoparticles has significantly improved their Cr(VI) adsorption capacity [24,25,26,27,28].

In this study, an innovative approach was employed to develop three types of nano adsorbents (composites, k-carrageenan-based composites, and chitosan-based nanocomposites), which originated from waste materials such as eggshells and fly ash. To the best of our knowledge, this is the first study reporting the simultaneous harnessing of the unique properties of both biopolymers (k-carrageenan and chitosan) alongside fly ash, zeolites, and eggshells for achieving highly efficient chromium removal from aqueous solutions. The study employed various analytical techniques, including the Brunauer–Emmett–Teller (BET) method, X-ray diffraction (XRD), Fourier-transform infrared (FTIR) spectroscopy, vibrating sample magnetometer (VSM), scanning electron microscopy (SEM), and thermogravimetric analysis (DTG), to assess the physical and chemical properties of each adsorbent type. Furthermore, this study delves into the intricacies of the adsorption mechanisms by rigorously analyzing adsorption isotherm models, thermodynamic properties, and adsorption and desorption kinetics. This multifaceted approach, complemented by after-adsorption analyses using FTIR, SEM-EDX, and thermal assessments, provides a deeper understanding of the adsorption mechanism. The investigation into the reusability of each adsorbent further emphasizes the sustainability of this research, demonstrating its potential for practical applications in environmental remediation. This study not only tackles the critical issue of heavy metal contamination in wastewater but also contributes to the valorization of agricultural and industrial waste, fostering a circular economy in environmental management.

## 2. Materials and Methods

### 2.1. Materials

All reagents used in this study were of analytical grade and sourced from commercial suppliers, including Merck (New York, NY, USA), Alfa Aesar (Haverhill, MA, USA), and Sigma-Aldrich (St. Louis, MO, USA), and were utilized without further purification. Kappa-carrageenan powder was obtained from Thermo Scientific (Tokyo, Japan), while chitosan powder (molecular weight range: 100,000–300,000) was acquired from Acros Organics (Geel, Belgium).

Eggshells (ESs) were sourced domestically and subjected to a procedure described in our previous paper [22,29].

Zeolite, obtained from Bentonita (Mediesu Aurit, Satu Mare, Romania), was prepared according to a procedure described in our previous papers [22,29].

Industrial-grade magnetite nanoparticles (average size: ~50 nm), purchased from Jalutex (Pucioasa, Romania), were then subject to a procedure described in our previous paper [29].

Electrofilter fly ash (average nanoparticle size: 25–55 nm) with a phase composition including Al_2_SiO_5_ (dialuminium silicate oxide), SiO_2_ (quartz), Al_2_O_3_ (corundum), and Ca_2_MgSi_2_O_7_ (akermanite) was provided by Colterm Cogeneration Power Station in Timisoara, Romania [12,29].

Composite materials referred to as FMZ (composed of fly ash, magnetite, and zeolite) and EMZ (comprising eggshells, magnetite, and zeolite) were prepared according to the methodology detailed below. Chromium (VI) solution with different concentrations (1 to 40 mg/L) was prepared from a stock solution of potassium dichromate (Merck, Darmstadt, Germany), which was dissolved in ultrapure water and then diluted to the desired final concentrations. For pH adjustment, 1 M HNO_3_ or NaOH solutions were used.

### 2.2. Instrumentations

The phase composition of the prepared adsorbents and their components was analyzed using a Bruker AXS D8-Advance powder X-ray diffractometer, utilizing CuKa radiation (λ = 0.1541 nm). The mean crystallite size was calculated employing the whole powder pattern fitting (WPPF) method.

Fourier-transform infrared (FT-IR) spectra of all prepared adsorbents and their solid-phase components were recorded using a Shimadzu IRTracer 100 FT-IR spectrophotometer (Columbia, MD, USA) with the Attenuated Total Reflectance (ATR) technique. The data collection process included 20 consecutive scans, which were performed at a resolution of 4 cm^−1^ over the spectral range of 4000 to 400 cm^−1^.

The surface area of the prepared adsorbents and their corresponding biopolymer matrices was evaluated utilizing multi-point Brunauer–Emmett–Teller (BET) regression within a relative pressure range of 0.08 to 0.3, employing a Nova 1200e high-speed surface area and porosity analyzer (Quantachrome, Boynton Beach, FL, USA). The specific surface area was computed according to BET theory. BET method (employing multi-point regression within the specified relative pressure range) and the Barrett-Joyner-Halenda (BJH) technique were used to determine the surface areas and the pore size distributions. Additionally, the total pore volume was ascertained from the final point of the isotherm, which yielded a value close to 1. All measurements were conducted in triplicate.

Magnetization properties of the adsorbents were evaluated using a Lake Shore 7410 vibrating sample magnetometer (VSM) (Westerville, OH, USA).

Thermal stability was assessed with a Mettler Toledo TGA/DSC3+ STARe System, performing thermal analyses between 25 °C and 400 °C in a dynamic air atmosphere (20 mL/min) at a heating rate of 10 °C/min, using 40 μL aluminum melting crucibles. Differential scanning calorimetry (DSC) was conducted under identical conditions.

Morpho-structural characterization of adsorbents was performed using an SEM-EDS system (JSM-IT200 InTouchScope™ Scanning Electron Microscope, Freising, Germany) equipped with a field emission gun (FEG). A Jaluba SW23 thermal shaker facilitated the batch adsorption experiments, while a Fritsch Pulverisette planetary mill was utilized in the preparation of the composite nanomaterials (FMZ and EMZ).

### 2.3. Preparation of Adsorbents

#### 2.3.1. FMZ Nanoadsorbent

The FMZ nanocomposite was prepared by blending fly ash, magnetite nanoparticles, and zeolite in a mass ratio of 1:1:1. This mixture was mechanically milled at a rotation speed of 500 rpm for 30 min at room temperature.

#### 2.3.2. EMZ Nanoadsorbent

The EMZ nanoadsorbent was prepared analogously, using a combination of eggshell, magnetite, and zeolite at the same mass ratio.

#### 2.3.3. Preparation of k-Carrageenan-Based Nanocomposites (FMZCa and EMZCa)

To prepare the k-carrageenan-based nanocomposites, 2 g of k-carrageenan was dissolved in 100 mL of ultrapure water and heated to 80 °C ± 2 °C for 2 h until the solution reached a concentrated state. Upon cooling to a temperature range of 60 °C ± 3 °C, to form a gel [30].

Subsequently, 2 g of the respective nanocomposite (FMZ or EMZ) was added and mixed thoroughly for 15 min. Following complete homogenization, this mixture was added dropwise to 25 mL of 3% KCl (potassium chloride) solution under stirring to form small beads (or pellets), filtered, and washed with ultrapure water. Each experiment was conducted in triplicate.

#### 2.3.4. Preparation of Chitosan-Based Nanocomposites (FMZC and EMZC)

Chitosan solution was formulated by dissolving 1.4 g of chitosan in 100 mL of a 5% (*v*/*v*) acetic acid solution, incubating this mixture at 50 °C for 2 h with continuous stirring. Following this, 0.25 g of the prepared nanocomposite (FMZ or EMZ) was incorporated into 18 mL of the chitosan solution at 40 °C ± 2 °C and stirred for 20 min. This resultant mixture was subsequently added dropwise to 25 mL of 2 M NaOH solution, filtered, and washed with deionized water. This experiment was also prepared in triplicate.

Before analyzing their properties, all biopolymer-based composites were dried in an oven at 50 °C for six hours.

**Figure 1:**
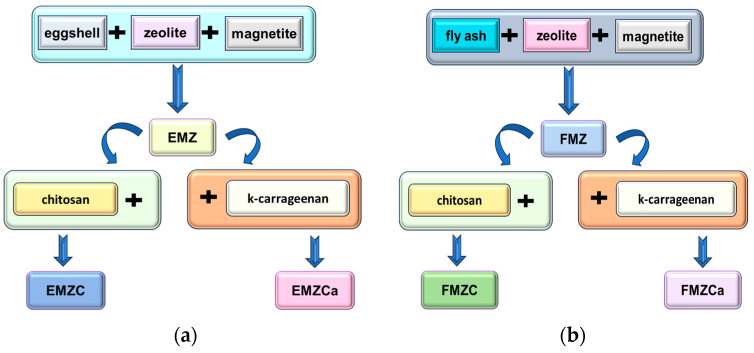
Schematic representation of composites, k-carrageenan-based nanocomposites, and chitosan-based nanocomposite preparation: EMZ/EMZC/EMZCa (**a**), and FMZ/FMZC/FMZCa (**b**).

A flowchart depicting the preparation of composites (EMZ and FMZ), k-carrageenan-based nanocomposites (EMZCa and FMZCa), and chitosan-based nanocomposites (EMZC and FMZC) is presented in Figure 1.

### 2.4. Batch Adsorption Study

The adsorption characteristics and mechanisms of FMZ, EMZ, k-carrageenan-based nanocomposites (FMZCa and EMZCa), and chitosan-based nanocomposites (FMZC and EMZC) were systematically analyzed through a series of isotherm, thermodynamic, and kinetic models.

#### 2.4.1. Evaluation of the Effects of Different Parameters on Adsorption

Different parameters were evaluated to determine the efficiency of heavy metal removal. The investigations considered the influence of various factors, including adsorbent dosage (0.50–3.5 g), contact time (0–480 min), pH (1.5–9.0), initial concentration of Cr(VI) (1–40 mg/L), and temperature (0–50 °C) on the performance of chromium adsorption.

Batch experiments were conducted in 100 mL Erlenmeyer flasks, each containing 50 mL of a heavy metal ion solution with a predetermined concentration. The flasks were maintained at 23.5 °C on a thermostat shaker operating at 200 rpm, with the variables of pH, adsorbent mass, and experimental temperature controlled until equilibrium was reached. Following the equilibrium period, the adsorbent was isolated through centrifugation and subsequent filtration using Whatman filter paper (0.45 μm). The chromium concentration in the filtrate was determined through atomic absorption spectrophotometry (Agilent 280FS, Santa Clara, CA, USA).

Each experimental condition was replicated three times, yielding results with an accuracy of ±2%.

The quantity of Cr(VI) uptake by the adsorbent at equilibrium (Q_e_, mg/g) was calculated using the following equation (Equation (1)):

(1) $$Q_{e}=\frac{\left(C_{e}-C_{o}\right)V}{m}$$

Removal efficiency (Re%) was computed as follows (Equation (2)):

(2) $$R_{e}=\frac{\left(C_{e}-C_{o}\right)}{C_{o}}\;100$$

where V = volume of the solution (mL)

m = dry adsorbent weight (g)

C_o_ and C_e_ represent initial and equilibrium concentrations of chromium (mg/L), respectively.

#### 2.4.2. Adsorbent Performance

The performance of each prepared adsorbent was assessed concerning its components over varying contact times. The experimental setup involved 100 mL Erlenmeyer flasks containing 2.00 g of adsorbent with a fixed volume of nickel solution (50 mL; 28.5 mg/L) at pH 4.5, maintained at room temperature (23 °C) and agitated at 180 rpm. Samples were collected at designated intervals (0–480 min), subjected to centrifugation, and filtered through Whatman filter paper (0.45 μm), followed by analysis of residual chromium concentration via atomic absorption spectrophotometry.

#### 2.4.3. Desorption Study

Experiments were conducted by incubating samples at a temperature of 23 °C. A constant volume (50 mL) of chromium solution (28.5 mg/L) was mixed with a fixed amount (2.00 g) of adsorbents and 15 mL of one of three desorption solutions (0.01 M HNO_3_, 0.01 M H_2_SO_4_, or 0.01 M EDTA). The resulting mixtures were stirred at a rate of 180 rpm, and aliquots were collected at 30 min intervals over the experiment duration (0–300 min). Following the collection process, the samples underwent centrifugation and were filtered using Whatman filter paper (0.45 μm). The amount of desorbed heavy metal was quantified utilizing atomic absorption spectrophotometry. The desorption rate was calculated using the next equation (Equation (3)):

(3) $$D=\frac{C_{d}}{C_{a}}\;100$$

C_d_ and C_a_ represent the concentration of chromium desorbed and retained, respectively.

#### 2.4.4. Kinetic Studies

Kinetic experiments were conducted under control conditions at a temperature of 25 °C and a pH of 4.5, utilizing 2.00 g of adsorbent in a 50 mL chromium solution with a concentration of 28.5 mg/L. Samples were collected at various intervals ranging from 0 to 400 min.

#### 2.4.5. Thermodynamic Study

The thermodynamic analysis was performed at three distinct temperatures: 283.15 K, 298.15 K, and 313.15 K, at pH 4.5 using a fixed amount of adsorbent (2.00 g) and constant volume of chromium stock solution (50 mL; 28.5 mg/L). The adsorption isotherms were represented graphically by plotting ln K (x-axis) against 1/T (y-axis). The correlation coefficients obtained were notably high, with R^2^ values of 0.986 for FMZ and 0.994 for EMZ, 0.989 for FMZCa, 0.991 for EMZCa, 0.995 for FMZ, and 0.998 for EMZC, indicating a good linear relationship in the derived data.

#### 2.4.6. Statistical Analysis

All experimental trials were performed in triplicate, and statistical significance was evaluated using one-way ANOVA without replication, with a significance threshold set at *p* < 0.05. BET analysis was carried out using the statistical test.

## 3. Results

### 3.1. BET Analysis

**Figure 2:**
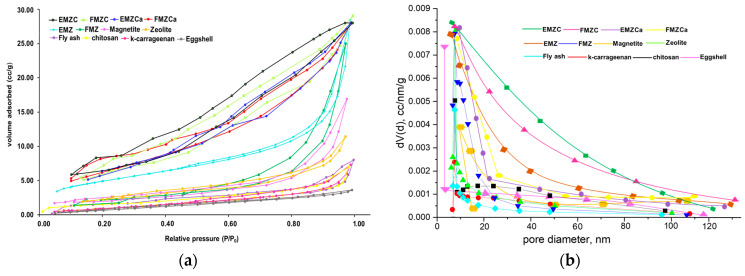
The nitrogen adsorption–desorption isotherms (**a**); pore distribution (**b**) for all adsorbents.

The surface properties of the synthesized adsorbents, which include nanocomposites (FMZ and EMZ), k-carrageenan-based nanocomposites (FMZCa and EMZCa), and chitosan-based nanocomposites (FMZC and EMZC), as well as their corresponding components, were evaluated using low-temperature (77 K) nitrogen adsorption–desorption isotherms (Figure 2).

The determination of surface areas and pore size distributions was conducted using the Brunauer–Emmett–Teller (BET) along with the Barrett–Joyner–Halenda (BJH) method. The results are summarized in Table 1.

The data revealed that the BET/N_2_ specific surface area values for eggshell (1.328 m^2^/g), magnetite (18.02 m^2^/g), zeolite (12.126 m^2^/g), and fly ash (18.02 m^2^/g) are consistent with previous literature and our prior studies. In contrast, the specific surface area values for the synthesized nanocomposites, EMZ and FMZ, demonstrated considerable variation, which was anticipated due to their differing compositions. Further analysis showed a significant increase in the surface area of the FMZ and EMZ nanocomposites compared to their raw components, indicating a notable advancement in material design for chromium adsorption applications. However, this enhancement was accompanied by a reduction in average pore diameter and total pore volume relative to zeolite, attributed to the eggshell particles in EMZ and fly ash in FMZ [29,31].

BET/N_2_ specific surface values for polymeric matrices, k-carrageenan (129.86 m^2^/g), and chitosan (42.77 m^2^/g) were consistent with values reported in the literature [32,33,34].

Notably, significant differences were identified in the specific surface areas of k-carrageenan-based nanocomposites (FMZCa and EMZCa) and chitosan-based nanocomposites (FMZC and EMZC) when compared to their respective components and biopolymer matrices. For the k-carrageenan-based nanocomposites, surface areas ranged from 142.77 m^2^/g for FMZ to 145.34 m^2^/g for EMZ, with average pore volumes and sizes measuring 11.02 nm and 13.88 nm, respectively. In the case of the chitosan-based nanocomposites, the surface area values varied from 192.12 m^2^/g (FMZC) to 201.83 m^2^/g (EMZC), accompanied by average pore volumes and sizes of 12.83 nm and 14.89 nm, respectively. These concurrent changes provide substantial versatility in selecting appropriate materials for specific applications.

As illustrated in Figure 2, the isotherms for chitosan-based nanocomposites (EMZC and FMZC) and k-carrageenan-based composites (EMZCa and FMZCa), as well as the composites (EMZ and FMZ), conform to a Type II isotherm characterized by an H2 hysteresis loop. This behavior indicates the presence of a macroporous structure within these materials. The porous architecture enhances the efficiency of pollutant immobilization and release.

### 3.2. XRD Study

**Figure 3:**
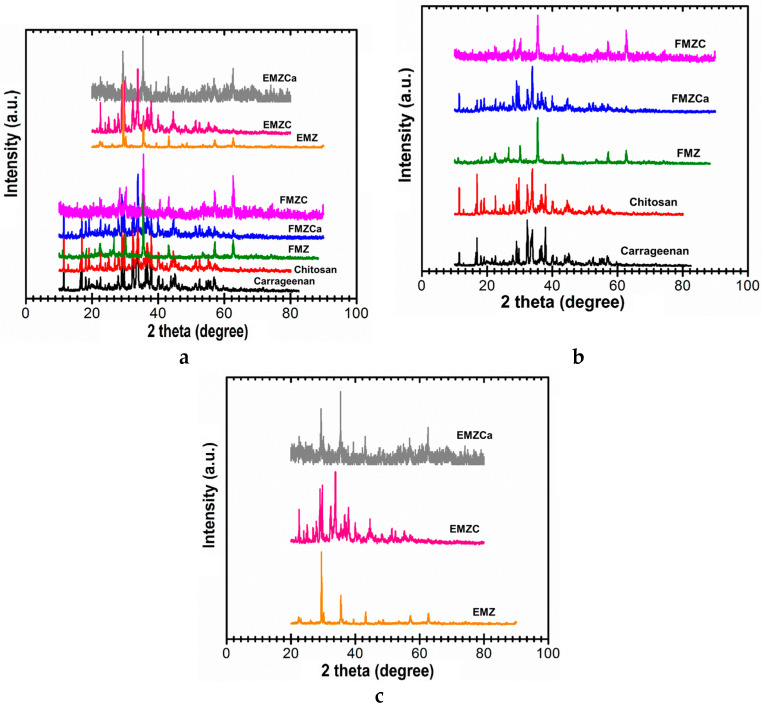
XRD spectra of all adsorbent types prepared and their components (**a**), chitosan, carrageenan, FMZ, FMZCa, FMZC (**b**), and EMZ, EMZCa and EMZC (**c**).

The X-ray diffraction (XRD) analysis of the FMZ sample, as depicted in Figure 3a,b, demonstrates the presence of well-preserved crystalline phases. Notably, these phases include magnetite (Fe_3_O_4_, COD 9005837), which exhibits sharp diffraction peaks at approximately 30°, 35°, 43°, and 57° [29]. The zeolite component, specifically clinoptilolite-Ca (COD 9001509), reveals characteristic peaks (~20°–30°). Additionally, the analysis identifies components associated with fly ash, which correspond to Al_2_SiO_5_ (COD 1010329), SiO_2_ (quartz, COD 9009666), Al_2_O_3_ (corundum, COD 1010951), and Ca_2_MgSi_2_O_7_ (akermanite, COD 9006450), along with a broad amorphous background (~15°–25°) associated with the amorphous fly ash component. All collectively affirming the successful integration and structural integrity of FMZ composite [22,29].

In the XRD spectrum of EMZ (Figure 3a,c) sharp peaks are visible with distinct contributions at ~23°, ~29°, ~36°, and ~47° from eggshell-derived calcite phase (CaCO_3_, COD 9000965), representing the dominant crystalline phase in eggshell [22,29]. Also, similar to FMZ, exhibit peaks at ~30°, ~35°, ~43°, and ~57° confirm the presence of magnetite as a significant phase [29]. Furthermore, the zeolite diffraction peaks appear in the range of ~20°–30°, confirming the retention of its crystalline structure during EMZ composite formation [22]. Notably, both FMZ and EMZ display amorphous characteristics, evidenced by broad bands across the 10–70 ° scanning interval, underscoring their nature as effective absorbents [22,29]. Nonetheless, compared to FMZ, EMZ appears to have a slightly lower amorphous content, reflecting the higher crystallinity of the eggshell component compared to the amorphous phase associated with fly ash in FMZ [22,29].

The X-ray diffraction (XRD) pattern for FMZCa demonstrates a reduction in peak intensity for magnetite, quartz, and zeolite, indicating a stronger encapsulation effect by k-carrageenan [35]. Additionally, the presence of the biopolymer matrix enhances the amorphous nature of FMZCa, as evidenced by a noticeable elevation in the baseline and the observation of broader, less-defined peaks within the XRD pattern. This indicates that the presence of the biopolymer matrix contributes to a more amorphous structure [35]. When comparing the k-carrageenan pattern, illustrated by the black line in Figure 3a,b, which exhibits broader and less intense peaks in the 2θ range of approximately 15°–25°, the FMZCa pattern presented by the blue line reveals sharper and more pronounced peaks. This finding implies that the encapsulation of FMZ into the κ-carrageenan matrix has been successfully achieved [35]. Moreover, the relatively smooth and consistent intensity peaks observed for FMZCa indicate uniform dispersion of the fly ash, magnetite, and zeolite particles within the k-carrageenan matrix, which is essential for maintaining the uniformity and functionality of FMZCa, thereby underscoring the success of its synthesis [35].

The X-ray diffraction (XRD) pattern of EMZCa (as shown in Figure 3a,c) indicates a notable decrease in crystallinity compared to the EMZ sample. This reduction is primarily attributed to the incorporation of k-carrageenan, which introduces an amorphous hump in the XRD pattern within the 2θ range of approximately 15° to 25° [35]. This feature is characteristic of this polysaccharide matrix and highlights the amorphous nature of k-carrageenan [35]. Despite the overall decrease in crystallinity, the primary peaks corresponding to magnetite, eggshell, and zeolite are still identifiable, albeit with diminished intensity. This observation suggests these components maintain structural integrity following EMZ incorporation into the k-carrageenan matrix [35]. Specifically, the magnetite peaks, which appear at approximately 2θ values of 30°, 35°, and 43°, remain visible but show a reduction in intensity. The peaks associated with the eggshell and zeolite components are also partially obscured due to the amorphous contribution from k-carrageenan [35].

The XRD pattern for chitosan (red line, Figure 3a,b) reveals a broad peak in the 2θ range of approximately 10°–25°, indicating the semi-crystalline nature arising from the partial alignment of its polymer chains in crystalline domains. The low intensity and lack of sharp peaks reflect the dominance of amorphous regions, a characteristic of biopolymers like chitosan [26].

The X-ray diffraction (XRD) analysis of the FMZC (Figure 3a,b) composite reveals distinct sharp and intense peaks attributed to the presence of crystalline phases, specifically magnetite (Fe_3_O_4_) (observed around 2θ = ~30°, ~35°, ~43°, and ~57) and zeolite (in the 2θ range of ~20°–30°), alongside contributions from fly ash (at 20°–40° range), superimposed on a relatively less prominent background from semi-crystalline chitosan matrix. These sharp peaks in the XRD pattern suggest the effective dispersion and incorporation of these crystalline materials within the amorphous chitosan matrix [36]. However, the slight broadening and reduction in the intensity of peaks, particularly those associated with zeolite and quartz, suggest encapsulation of these particles within the chitosan matrix [26,36]. Additionally, the partial masking of the fly ash peaks further supports the notion that the crystalline phases are embedded within the amorphous chitosan. Overall, the crystallinity of the FMZC composite is enhanced by these crystalline components incorporation while the chitosan matrix plays a crucial role in modulating the FMZ structural characteristics and properties.

Similarly, the EMZC pattern (Figure 3a,c) exhibits a slight reduction in the intensity of the peaks associated with magnetite, zeolite, and eggshell compared to the EMZ pattern. This observation suggests the encapsulation of EMZ within the chitosan matrix [26,36]. Furthermore, the notable absence of prominent peaks corresponding to chitosan in the EMZC pattern indicates that the crystalline characteristics of the EMZ dominate the overall composite diffraction pattern. Although the EMZ incorporation of the chitosan matrix introduces some reduction in crystallinity, most of the structural peaks associated with the EMZ remain preserved.

The X-ray diffraction (XRD) analysis demonstrates a reduction in crystallinity and a pronounced decrease in the characteristic chitosan peak at 2θ ≈ 20.3°, confirming the successful synthesis of chitosan-based nanocomposites. A comparative evaluation of the XRD patterns for FMZ, FMZCa, and FMZC (Figure 3a,b), as well as EMZ, EMZCa, and EMZC (Figure 3a,c), indicates that the varying crystallinity of these materials results in overlapping patterns where only the most intense peaks remain visible. The biopolymer-based nanocomposites exhibit a semicrystalline character, further validating their effective formation.

Overall, the XRD analysis provides valuable insights into the structural properties of EMZ and FMZ composites, highlighting the significant impact of chitosan and k-carrageenan matrices on their crystallinity. While the inorganic components—magnetite, zeolite, and eggshell or fly ash—retain their crystalline structure during composite formation, interactions with the biopolymer matrices lead to notable structural modifications. The semi-crystalline nature of chitosan and the predominantly amorphous character of k-carrageenan interact with the inorganic phases, causing peak broadening and intensity reduction [35,36]. These observations point to substantial changes in the dispersion and arrangement of inorganic particles within the polymeric matrix, which ultimately influence the mechanical, physical, and functional properties of the newly developed biopolymer-based nanocomposites.

### 3.3. SEM Analysis

**Figure 4:**
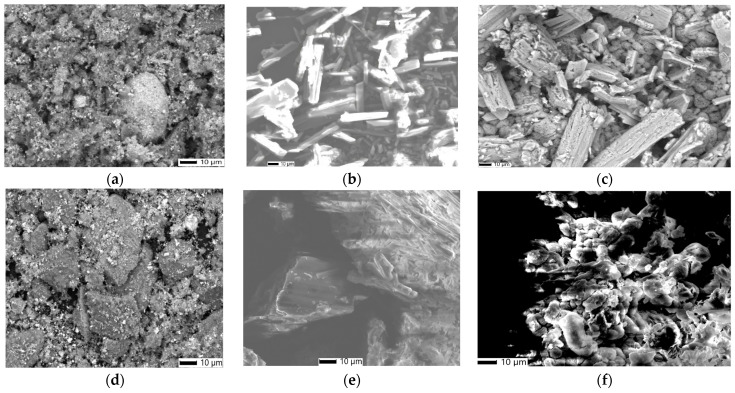
SEM images of FZM (**a**), FMZCa (**b**), FMZC (**c**), EMZ (**d**), EMZCa (**e**), and EMZC (**f**).

The surface morphology, shape, and particle size of all proposed nano adsorbents (FMZ, EMZ, FMZCa, EMZCa, FMZC, and EMZC) were studied using SEM (Figure 4).

The FMZ micrographs (Figure 4a) reveal clusters of nanoparticles with varying sizes in the nanometer range, specifically, spherical particles (~10 nm) derived from fly ash and magnetite cubic-shaped particles (~18 nm) interspersed within rectangular crystal structures typical of zeolite [22,29,37]. These particles are both present on the surface and within the pores of the zeolite matrix. Similarly, the EMZ micrograph (Figure 4d) demonstrates a comparable distribution of magnetite cubic particles loaded in zeolite and eggshell-like pores.

When examining the k-carrageenan-based nanocomposites (FMZCa (Figure 4b) and EMZCa (Figure 4e), notable morphological distinctions arise compared to FMZ and EMZ, particularly in terms of reduced cluster sizes. The FMZCa micrograph (Figure 4b) displays a variety of spherical and rectangular structures (averaging 12 nm) that are assembled and uniformly dispersed within the carrageenan matrix alongside carrageenan fibers [38].

Conversely, the EMZCa (Figure 4e) micrograph features an assortment of rectangular and cubic nanoparticles dispersed throughout the carrageenan matrix.

The prepared chitosan-based nanocomposite samples (FMZC and EMZC) exhibit the following characteristics:

(a) The FMZC micrograph (Figure 4c) illustrates a uniform distribution of smoother spherical, cubic, and rectangular particles uniformly dispersed in the polymeric matrix.
(b) The EMZC micrograph (Figure 4f) reveals a consistent distribution of rectangular and cubic particles within the organic matrix [39].

All biopolymer-based nanocomposites (FMZCa, EMZCa, FMZC, and EMZC) highlight a significant reduction in cluster sizes; they measure around 35 nm for chitosan-based nanocomposites and around 40 nm for k-carrageenan-based nanocomposites.

### 3.4. FTIR Analysis

**Figure 5:**
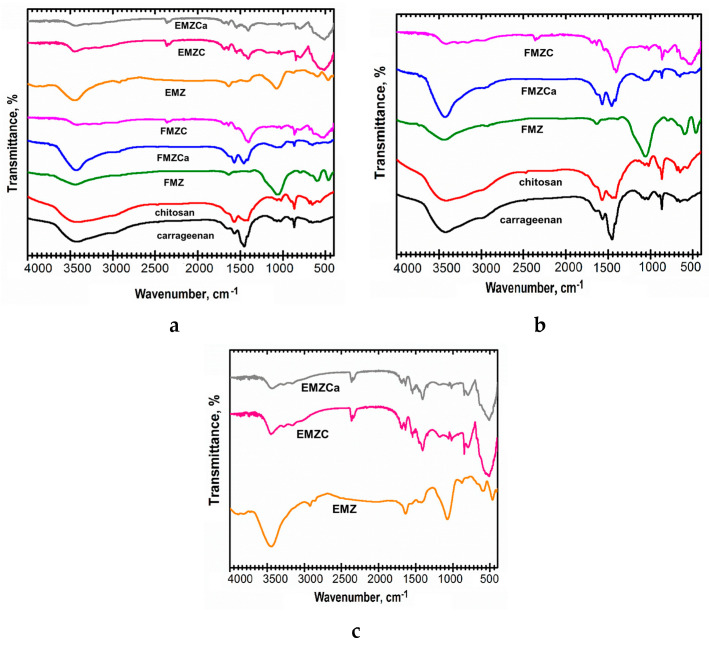
FT-IR spectra of all adsorbent types prepared and their components (**a**), chitosan, carrageenan, FMZ, FMZCa, and FMZC (**b**), EMZ, EMZCa and EMZC (**c**).

FT-IR spectra were recorded for EMZ, FMZ, EMZCa, FMZCa, EMZC, and FMZC, and polymeric matrices (carrageenan and chitosan) (Figure 5).

The FT-IR spectra and XRD patterns of starting materials for adsorbents (eggshell, fly ash, magnetite, and zeolite) are similar since they come from identical sources, as reported in our previous sources [12,22,29].

The spectrum for FMZ (Figure 5a,b) exhibits the magnetite vibrational bands at ~597 cm^−1^ associated with the (Fe-O) [40], the peaks attributed to zeolite at 464 cm^−1^ (Si-O-Si), ~1066 cm^−1^ (Si-O asymmetric stretch). Vibrational peaks assigned to fly ash were found at ~588 cm^−1^ (Ca-O), ~ 670 cm^−1^ (Al-O-Al bending vibration), and 830 cm^−1^ (AlO_4_ coordination) [22,41,42,43]. The large band at 3440 cm^−1^ indicates the presence of moisture and water molecules [22,41,44,45].

The FTIR spectra of EMZ (Figure 5a,c) display the vibrational bands of the characteristic peaks associated with eggshell at ~714 cm^−1^ (Ca-O stretch), ~880 and ~1418 cm^−1^ (C-O stretch), 1637 cm^−1^ (C=O stretching vibrations), and 1643 cm^−1^ (assigned to N-H), 1077 cm^−1^ (C-N stretching) [22,41,44,45]. The peaks assigned to magnetite were found at ~592 cm^−1^ associated with the (Fe-O) [40]. The peaks attributed to zeolite at 469 cm^−1^ (Si-O-Si), ~1066 cm^−1^ (Si-O asymmetric stretch). The presence of moisture and water molecules is associated with the large band at 3445 [22,41,44,46,47].

Regarding the k-carrageenan-based nanocomposites, the EMZCa spectra (Figure 5a,c) depicted the vibrational peaks from EMZ and carrageenan (Figure 5a). Nonetheless, a series of notable differences (intensity of vibrational peaks and displacement of k-carrageenan absorption bands in the region of sulfate ester region (1454, 1394, 1266, 848, and 705 cm^−1^) indicate interactions between polymer and EMZ functional groups. Shifting the intensity of the peaks in the sulfate ester region and displacement to longer wavelengths can be observed, indicating interactions between the polymer and the functional groups in the EMZ.

The FT-IR spectra of FMZCa (Figure 5a,b) show the discriminative peaks of FMZ and carrageenan with a slight shifting in the intensity of the peaks and displacement to longer wavelengths in the sulfate ester region, supporting the formation of FMZCa.

Following EMZ encapsulation, in the EMZC spectra (Figure 5a,c), all the characteristic peaks of chitosan (Figure 5a) (~1568, ~1436, ~1413, ~1077, ~1024, ~869, ~650, and ~565 cm^−1^) and the EMZ adsorbent were observed. However, several intensity changes and small shifts in the N-H (1568, 1018, and 870 cm^−1^) can be attributed to the interaction between ammonium groups of chitosan interaction with functional groups of EMZ.

Similarly, the FMZC spectra (Figure 5a,b) show all the characteristic vibrational bands from chitosan and FMZ, and notable changes appear in the same N-H region, indicating the formation of interaction between ammonium groups of chitosan interaction with functional groups of FMZ.

### 3.5. Magnetic Measurements

**Figure 6:**
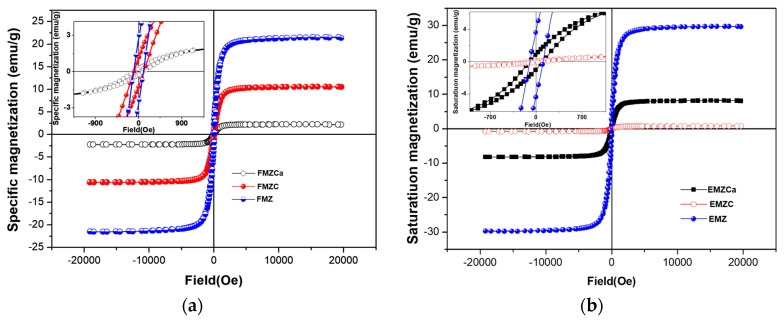
VSM of FMZ:FMZCa:FMZC (**a**) and EMZ:EMZCa:EMZC (**b**).

A vibrating sample magnetometer (VSM) was employed to assess the saturation mass magnetization of adsorbents possessing the same composition. The vibrating VSM loops (Figure 4) indicated a predominantly ferromagnetic profile for the samples, with the notable exceptions of FMZCa and EMZC, which displayed a hysteretic profile exhibiting paramagnetic behavior near zero fields (Figure 6 and Table 2).

The variations in saturation magnetization among the different types of prepared adsorbent, specifically nanocomposites (FMZ, EMZ), k-carrageenan-based nanocomposites (FMZCa and EMZCa), and chitosan-based nanocomposites (FMZC and EMZC) are directly correlated with the content of magnetic materials and the experimental methodologies utilized in this study. It is important to note that the magnetic properties of the magnetite nanoparticles remain unchanged throughout the experimental processes; consequently, the specific magnetization of the k-carrageenan-based and chitosan-based nanocomposites is determined solely by the mass ratios of the magnetic materials to the polymeric matrices.

### 3.6. Thermal Analysis

**Figure 7:**
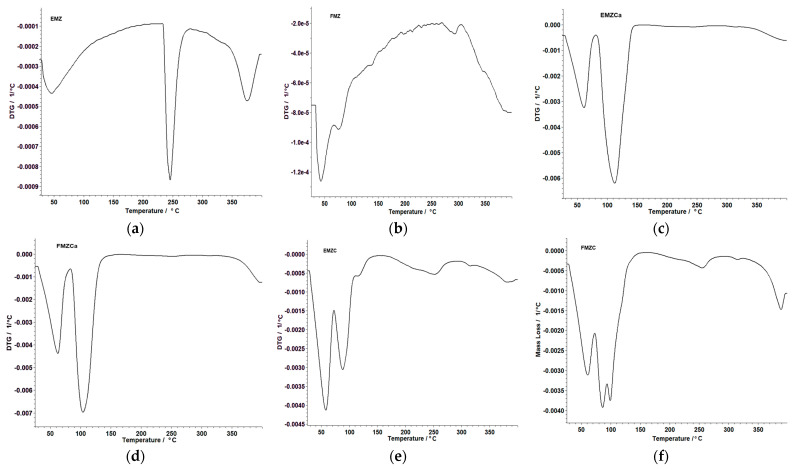
TGA thermograms of EMZ (**a**), FMZ (**b**), EMZCa (**c**), FMZCa (**d**), EMZC (**e**), and FMZC (**f**).

The thermal behavior of the prepared adsorbents was studied to assess their stability concerning their material types, specifically, nanocomposites, kappa-carrageenan-based nanocomposites, and chitosan-based nanocomposites. The data obtained are presented in Figure 7 and Table 3.

The thermal analysis of all prepared adsorbents has revealed distinct stages of thermal decomposition. Notably, FMZ (Figure 7b), EMZCa (Figure 7c), and FMZCa (Figure 7d) exhibited two decomposition stages (Table 3), while EMZC (Figure 7e), FMZC (Figure 7f), and EMZ (Figure 7a) displayed three stages. In the case of EMZCa, the initial stage involved the loss of crystallization water, followed by the removal of water from the membrane matrix and the subsequent degradation of the polymer matrix (Table 3). Similarly, FMZCa and FMZ displayed analogous processes characterized by specific temperature ranges and associated weight losses for each stage (Table 3).

Conversely, the thermal decomposition of EMZC and FMZC (Fig unfolded through three distinct stages, encompassing moisture elimination, water removal from the membrane matrix, and the thermal degradation of the polymer matrix, each occurring at different temperature ranges with specific weight losses (Table 3).

The thermal decomposition of EMZ (Figure 7a, Table 3) occurs in three stages, encompassing moisture elimination, loss of crystallization water, and thermal degradation associated with organic components from eggshell particles. The analysis of the thermal decomposition of biopolymer-based nanocomposites unveiled differences in stability. Specifically, EMCa was found to exhibit more stability than FMZCa, and EMZC was determined to be more stable than FMZC. Additionally, FMZ was ascertained to be more stable than EMZ. Interestingly, notwithstanding the disparities in stability, encapsulation rendered EMZ more thermally stable than FMZ, irrespective of the biopolymer type used.

### 3.7. Adsorption Properties

#### 3.7.1. Effect of Adsorbent

**Figure 8:**
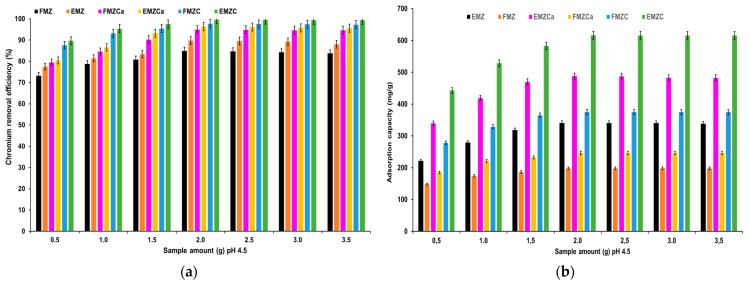
Chromium removal efficiency (**a**) and adsorption capacity (**b**) as a function of adsorbent mass.

The chromium removal efficiency and adsorption capacity of all prepared adsorbents (EMZ, FMZ, EMZCa, FMZCa, EMZC, and FMZ) were examined as a function of adsorbent mass (Figure 8).

The data depicted in the graphs within Figure 8 indicate a significant increase in chromium adsorption as the quantity of adsorbent is raised from 0.50 g to 2.0 g. Maximum adsorption is observed at 2.0 g of each prepared adsorbent, with values of 89.76% and 349.84 mg/g for EMZ; 84.83% and 198.47 mg/g for FMZ; 94.87% and 246.53 mg/g for FMZCa; 96.36% and 487.82 mg/g for EMZCa; 97.67% and 375.45 mg/g for EMZCa; 97.67% and 375.45 mg/g for FMZC; and 99.64% and 616.19 mg/g for EMZC, respectively. Following the attainment of equilibrium, adsorption exhibits a slight decline with further increases in adsorbent quantity. These findings suggest that a higher mass of adsorbent leads to a greater availability of active sites until equilibrium is attained. However, beyond that point, an increase in adsorbent mass leads to agglomeration, reducing the specific surface area and active sites [22,44,48].

#### 3.7.2. Effect of Initial Concentration on Chromium Removal Efficiency

**Figure 9:**
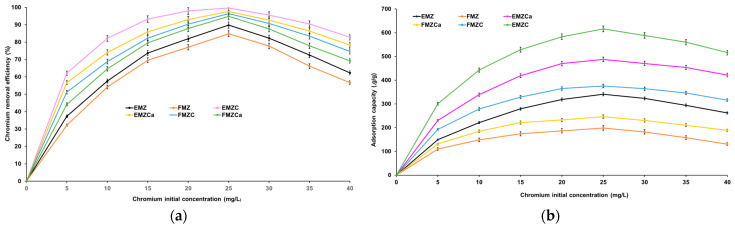
Relationship between chromium (**a**) initial concentration and chromium removal efficiency (%) and (**b**) initial concentration and adsorption capacity (mg/g).

The concentration of pollutants at the outset significantly influences the adsorption process. Therefore, were investigated the impact of the initial heavy metal concentration on chromium removal efficiency and adsorption capacity for each prepared adsorbent (Figure 9).

The efficiency of pollutant removal is directly proportional to the increase in the initial concentration of the pollutant within the range of 0–25 mg/L for all the prepared adsorbents (Figure 9). As depicted in Figure 9b, the adsorption capacities of all adsorbents exhibit a similar increasing trend with the initial pollutant concentration ranging from 0 to 25 mg/L, reaching maximum values of 349.84 mg/g for EMZ, 198.47 mg/g for FMZ, 246.53 mg/g for FMZCa, 487.82 mg/g for EMZCa, 375.45 mg/g for EMZC, and 616.19 mg/g for EMZC, respectively. The maximum removal efficiencies for FMZ (84.83%), EMZ (89.76%), FMZCa (94.87%), EMZCa (96.36%), FMZC (97.67%), and EMZC (99.64%) were achieved at Cr(VI) concentrations of 25.0 mg/L (Figure 9a). Beyond this point, all removal efficiencies exhibit a slightly decreasing trend, and the same pattern is observed for the adsorption capacities. In accordance with collision theory, these findings suggest that an increase in chromium concentration, and thereby the number of chromium ions, results in an accelerated reaction rate due to the increased potential for interaction with acceptor sites on all the prepared adsorbents (EMZ, FMZ, EMZCa, FMZCa, EMZC, and FMZC) until the equilibrium concentration is attained [49]. Subsequently, an imbalance between numerous chromium ions and a progressively decreasing number of active sites available on the adsorbents causes a decline in the adsorption potential for all the prepared adsorbents. These outcomes align with the data reported for the component materials of the adsorbents [50,51,52].

#### 3.7.3. Effect of pH

**Figure 10:**
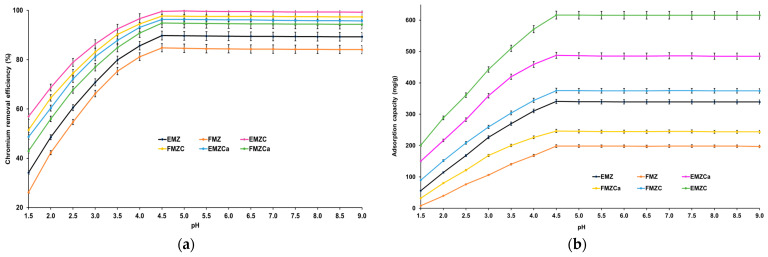
Relationship between chromium (**a**) pH and chromium removal efficiency (%) and (**b**) pH and adsorption capacity (mg/g).

The pH significantly affects the adsorption process by influencing the ionic chemical speciation of the adsorbing species and the adsorbent surface [21,40,42,52,53]. Given this information, the study investigated the adsorption of chromium ions on each prepared adsorbent as a function of pH. Figure 10 illustrates the relationship between pH and chromium removal efficiency, as well as adsorption capacity.

The data indicate that raising the pH level from 1.5 to 4.5 causes a notable increase in chromium ions adsorbed per unit mass of adsorbent. The maximum values for adsorption efficiency and capacity are reached at pH 4.5 (84.83% and 198.47 mg/g for FMZ; 89.76% and 349.84 mg/g for EMZ; 94.87% and 246.53 mg/g for FMZCa; 96.36% and 487.82 mg/g for EMZCa; 97.67% and 375.45 mg/g for FMZC; and 99.64% and 616.19 mg/g for EMZC), with no further changes later.

In an acidic environment, chromium in its hexavalent state (Cr(VI)) primarily exists as HCrO_4_^−^ and Cr_2_O_7_^2−^ ions, both of which carry a negative charge. In contrast, the surface of the adsorbent is generally positively charged. As a result, the increased adsorption observed at acidic pH can be attributed to the chemical interactions between chromium species and the adsorbent surface. At higher concentrations of Cr(VI), stable forms such as H_2_CrO_4_ and CrO_3_ can lead to the formation of polynuclear species, enhancing removal efficiencies at lower pH levels. Additionally, the abundance of H^+^ ions neutralizes the negatively charged surface of the adsorbent, facilitating the diffusion of dichromate ions. The adsorption process is further influenced by an electric double-layer formation at the adsorbent interface, which transitions from positive to negative as the concentration of H^+^ ions decreases with increasing pH.

Various studies have indicated that at lower pH levels, the system reaches equilibrium more quickly, resulting in a higher percentage of chromium uptake [1,5,54]. However, when the pH exceeds 7, the primary stable species shifts to CrO_4_^2−^, and the suppression of Cr(VI) hydrolysis can account for the observed reduction in adsorption capacity.

The emerging results align well with the previously reported data regarding the initial materials utilized for all types of adsorbent preparation [5,55,56,57,58].

#### 3.7.4. Effect of Contact Time

**Figure 11:**
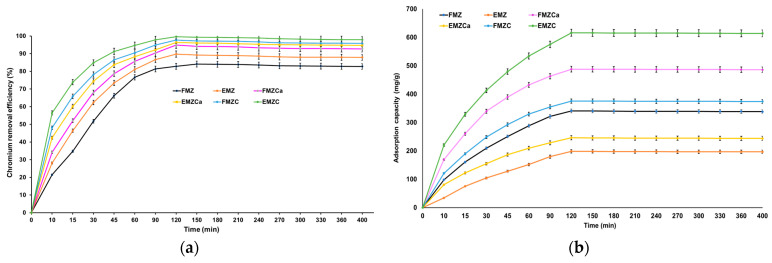
Relationship between chromium (**a**) time and chromium removal efficiency (%) and (**b**) time and adsorption capacity (mg/g).

The duration of adsorption plays a crucial role in influencing the uptake capacity of chromium ions. To determine the best contact time for maximizing adsorption capacity and pollutant removal efficiency, we examined the uptake capacities of all prepared adsorbents over varying contact times. The results, depicted in Figure 11, show that both removal efficiency and adsorption capacity increase as the contact time with the investigated adsorbents is extended.

Furthermore, equilibrium is achieved at 120 min, with maximum adsorption capacities of 349.84 mg/g for EMZ, 198.47 mg/g for FMZ, 246.53 mg/g for FMZCa, 487.82 mg/g for EMZCa, 375.45 mg/g for FMZC, and 616.19 mg/g for EMZC. Additionally, at this time point, nickel removal efficiency reaches its peak at 84.83% for FMZ, 89.76% for EMZ, 94.87% for FMZCa, 96.36% for EMZCa, 97.67% for FMZC, and 99.64% for EMZC. A more detailed examination of the data reveals three distinct stages in chromium adsorption:

(a) In the initial stage (0–120 min), adsorption increases rapidly, likely due to the high availability of active sites in the adsorbent.
(b) During the second stage (120–240 min), a notable decrease in the adsorption rate can be observed, attributed to the reduction in the number of available active sites.
(c) In the third stage (240–460 min), metal adsorption shows a plateau trend, indicating saturation of the active sites after reaching equilibrium. The data suggest that the optimal time required for the adsorption process to reach equilibrium for each investigated adsorbent is 120 min.

#### 3.7.5. Effect of Temperature on the Adsorption Process

**Figure 12:**
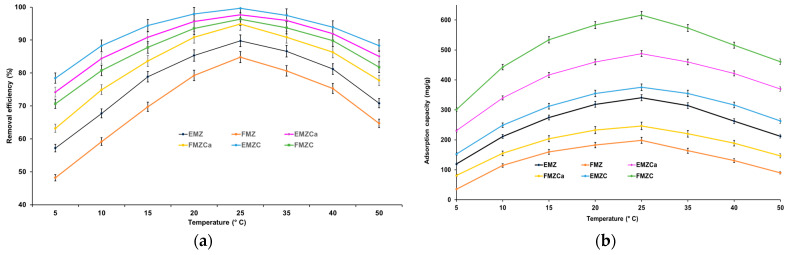
Relationship between chromium (**a**) temperature and chromium removal efficiency (%) and (**b**) temperature and adsorption capacity (mg/g).

The temperature plays a crucial role in the adsorption process, exerting a discernible influence on the efficacy of an adsorbent [40]. The impact of temperature on the adsorption process was examined across a temperature range of 5–50 °C was investigated for all prepared adsorbents (FMZ, EMZ, EMZCa, FMZCa, EMZC, and EMZC) (Figure 12).

The results revealed that adsorption is an endothermic process, with chromium removal efficiency and adsorption capacity increasing almost linearly with temperature up to a maximum, after which there is a slight decrease. The maximum removal efficiency (84.83% for FMZ, 89.76% for EMZ, 94.87% for FMZCa, 96.36% for EMZCa, 97.67% for FMZC, and 99.64% for EMZC) was observed at 25 °C, with corresponding maximum chromium adsorption capacities for all adsorbents (349.84 mg/g for EMZ, 198.47 mg/g for FMZ, 246.53 mg/g for FMZCa, 487.82 mg/g for EMZCa, 375.45 mg/g for FMZC, and 616.19 mg/g for EMZC). The data suggest that temperatures in the 5–25 °C range favor increased mobility of metal ions and interaction with acceptor sites on the adsorbent, leading to physical adsorption. At temperatures higher than 25 °C, a shift toward chemisorption is observed. Notably, even at 50 °C, high removal efficiency values (>77%) were obtained for all biopolymer-based-nanocomposites (77.77% for FMZCa, 85.09 for EMZCa, 88.34% for EMZC and 81.78 for FMZC), indicating that a temperature increase beyond 40 °C has minimal effect on the adsorption process, particularly in comparison to nanocomposites (64.73% for FMZ and 70.84% for EMZ).

#### 3.7.6. Chromium Removal Efficiency—Comparative Analysis Among All Prepared Adsorbent Types and Main Components

**Figure 13:**
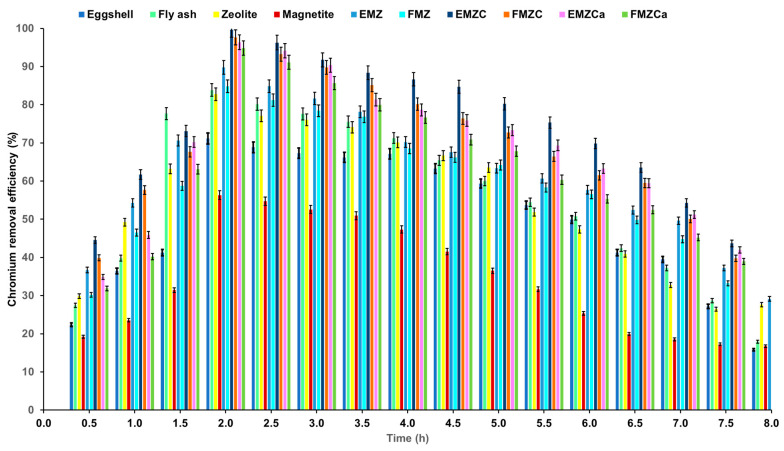
Removal efficiency and contact time relationship for all four adsorbents.

A comparative study was conducted to analyze the performance of adsorbents FMZ and EMZ before and after encapsulation in polymeric matrices (carrageenan (FMZCa, EMZCa) and chitosan (FMZC and EMZ) and raw materials (eggshell, fly ash, magnetite, and zeolite), concerning chromium removal efficiency over different contact times (Figure 13).

The results demonstrated a correlation between increased chromium removal efficiency and prolonged contact time for all adsorbents, culminating in peak efficiency at two hours, indicative of adsorption equilibrium. Subsequently, the adsorption rate diminished post-equilibrium. Notably, the experimental findings revealed a descending order of pollutant removal efficiency as follows: EMZ (99.64%) > FMZC (97.67%) > EMZCa (96.36%) > FMZCa (94.87%) > EMZ (89.76%) > zeolite (87.74%) > FMZ (84.83%) > fly ash (83.86%) > eggshell (71.14%) > magnetite (56.33%). These outcomes were consistent with the specific surface and porosity attributes of the adsorbents, as ascertained through BET analysis (Table 1). Furthermore, the adsorption efficiency values for eggshell, fly ash, magnetite, and zeolite closely corresponded with values reported in existing literature [29,44,58,59].

### 3.8. Adsorption Isotherms

Adsorption isotherms have been employed to examine the equilibrium partitioning of chromium between the adsorbent and solution. The Langmuir and Freundlich models are widely acknowledged as the most prevalent and dependable approaches for ascertaining the maximum chromium adsorption capacity through adsorption isotherms [52,60,61]. Several studies reported chromium adsorption on eggshells, magnetite, and zeolite adsorbents conform to Langmuir and Freundlich’s adsorption isotherms [41,52]. Furthermore, numerous studies have indicated that the adsorption isotherms of Cr (VI) on eggshell, magnetite, fly ash, and zeolite, derived using the mathematical equations of Temkin and Dubinin–Radushkevich (D-R) adsorption models, inadequately align with the experimental findings [22,29,52,62,63]

Consequently, the Langmuir and Freundlich adsorption isotherms were deemed suitable for developing an appropriate adsorption model capable of replicating this study’s experimental outcomes [60].

The Langmuir model is founded on the theoretical principles that (i) the adsorbent possesses a single, homogeneous layer in which the adsorption process occurs, and (ii) each of the adsorbed molecules exhibits identical adsorption energy without interaction between these molecules [60]. The linear representation of the Langmuir model is articulated by the following equation (Equation (4)):

(4) $$\frac{C_{e}}{Q_{e}}=\frac{1}{{K_{L}Q}_{m}}+C_{e}\;Q_{m}$$

where, $K_{L}$ (L/mg) is the Langmuir adsorption constant.

The Freundlich isotherm model is especially adequate for describing multilayer adsorption on heterogeneous surfaces, where non-uniform heat distribution and interactions between adsorbed molecules play a significant role [21,52]. The linear form of Freundlich isotherm is presented in Equation (5).

(5) $$logQ_{e}=log\;K_{F}+n\log\;C_{e}$$

where K_F_ (mg/g) and n (g/L) represent the Freundlich isotherm constants

In the case of chromium (VI) adsorption on the newly prepared adsorbents (FMZ, EMZ, EMZCa, FMZCa, EMZC, and FMZC), the Freundlich isotherm was applied to apprehend the complexity of adsorption dynamics considering the involvement of multiple layers or variable adsorption energies [61,64,65]. It has been utilized to depict the adsorption of Cr(VI) across all prepared adsorbents, taking into account the energy site distribution and competition interaction among different ions for available active sites from the adsorbents. The experimental results were plotted using the linear representation of Freundlich and Langmuir isotherm models and the determined parameters are shown in Table 4.

According to data from Table 4, both the Freundlich and Langmuir models have provided satisfactory descriptions of chromium absorption on the considered adsorbents. The calculated maximum adsorption capacity values align excellently with experimental equilibrium values for the newly prepared nanomaterials. Notably, the R^2^ value derived from the Freundlich model slightly surpasses that derived from the Langmuir model, indicative of a multimolecular layer adsorption process on irregular surfaces. Furthermore, the R_L_ values obtained from the Langmuir isotherm suggest a favorable adsorption process, falling within the range of 0 < RL < 1 for all prepared adsorbent types. Additionally, the values of the Freundlich constant n, indicative of the linearity of the adsorption, exceed 1, suggesting a suitable physical adsorption process on all investigated adsorbents’ heterogeneous surfaces [60,63,66].

### 3.9. Thermodynamic Study

The thermodynamic properties of all prepared adsorbents (EMZ, FMZ, EMZCa, FMZCa, EMZC, and FMZC) were systematically examined to evaluate their efficacy in chromium removal. This assessment encompassed the determination of Gibbs free energy (ΔG^0^), entropy (ΔS^0^), and enthalpy (ΔH) utilizing the Gibbs–Helmholtz and van’t Hoff equations [22].

(6) $$\Delta G^{0}=-RT\;lnK$$

(7) $$lnK=\frac{-\Delta H^{0}}{RT}+\frac{\Delta S^{0}}{R}$$

where,

R = 8.314 J/mol K

K (mL/g) = adsorption equilibrium constant

T (K) = the absolute temperature.

The investigations were conducted at three distinct temperatures (295.15 K, 303.15 K, and 313.15 K) using a 28.5 mg/mL chromium stock solution and maintaining a constant pH of 4.5. The slope and intercept of the van’t Hoff plot correspond to the thermodynamic parameters ΔG^0^ and ΔS^0^, as detailed in Table 5.

Negative ΔG^0^ values indicate the thermodynamic feasibility and spontaneity of the prepared adsorbents for chromium removal within the specified temperature range. The ΔH^0^ values (28.48 kJ/mol for EMZ, 21.41 kJ/mol for FMZ, 43.30 kJ/mol for EMZCa, 36.49 kJ/mol for FMZCa, 58.53 kJ/mol for EMZC and 47.56 kJ/mol for FMZC) suggest an endothermic adsorption process with a favorable affinity for chromium in all prepared adsorbents. Furthermore, positive ΔS^0^ values indicate that the adsorption process could involve structural changes and demonstrate the affinity of all prepared adsorbents (EMZ, FMZ, EMZCa, FMZCa, EMZC, and FMZC) for chromium ions [22,61,62,67].

### 3.10. Adsorption Kinetic Study

Various mechanisms, such as mass transfer, particle diffusion, diffusion control, and chemical reactions, can influence the adsorption process. Kinetic studies were conducted to assess the effectiveness of the adsorbent, gain insight into the mass transfer process, and determine the dynamic parameters of adsorption, including rate and temperature [61,68]. The experimental data on chromium adsorption for the newly prepared adsorbents (EMZ, FMZ, EMZCa, FMZCa, EMZC, and FMZC) were analyzed using a pseudo-first-order kinetic model, a pseudo-second-order kinetic model, and an intraparticle diffusion model. The pseudo-first-order kinetic model, known as the Lagergren equation, postulates that the adsorption rate is unforeseen upon the availability of active sites. The linear form of the Lagergren equation, represented as Equation (8), is employed to evaluate the adsorption process.

(8) $$ln\;(Q_{e}-Q_{t})=lnQ_{e}\;-\;K_{1}t$$

K_1_ = (min^−1^) is the rate constant.

The pseudo-second-order model assigns that the adsorption rate is contingent upon the presence of chemical interaction between heavy metal ions and the functional groups on the prepared adsorbents. The linearized form of second-order kinetics is delineated in the following equation (Equation (9)), where K_2_ [mg/(g min)] denotes the rate constant

(9) $$\frac{1}{Q_{t}}=\frac{1}{Q_{e}}t+\frac{1}{K_{2}Q_{e}^{2}}$$

The Weber and Morris intraparticle diffusion model postulates that the diffusion of chromium ions through adsorbent pores affects the adsorption rate and is expressed by Equation (10), with K*_i_* [mg/(g × min^−1/2^)] representing the intraparticle diffusion rate constant and C (mg/g) is a constant associated with the boundary layer thickness.

(10) $$Q_{t}{=K}_{i}t^{1/2}+C$$

The kinetic constants derived from the slope and intercepts of the kinetic models for chromium adsorption on all newly prepared adsorbents are shown in Table 6.

The analysis of the obtained results indicates negligible disparities between the correlation coefficients for the pseudo-first-order and pseudo-second-order kinetic models, suggesting that chromium adsorption on the adsorbents involves both physical and chemical processes. Additionally, it can be noted that the calculated adsorption capacities at equilibrium closely align with the experimental values obtained using the pseudo-second-order kinetics model, indicating that the adsorption of chromium ions predominantly relies on chemisorption, involving the formation of chemical bonds between chromium ions and active sites. The high correlation coefficient for the intraparticle diffusion model (exceeding 0.97 for all adsorbent types) suggests the involvement of intraparticle diffusion in the adsorption process. Nevertheless, intraparticle diffusion is not the sole rate-limiting step, as the diagram exhibits a non-linear pattern and deviates from the origin. These kinetic findings are consistent with the existing literature on the starting materials of all proposed adsorbent types.

### 3.11. Insight into Adsorption

The evaluation of structural and morphological changes was conducted through FT-IR, SEM-EDX, and thermal analysis techniques. This comprehensive approach aimed to investigate the potential mechanisms of chromium adsorption associated with the proposed nanomaterials and each type of biopolymer-based composite.

#### 3.11.1. FTIR Study

**Figure 14:**
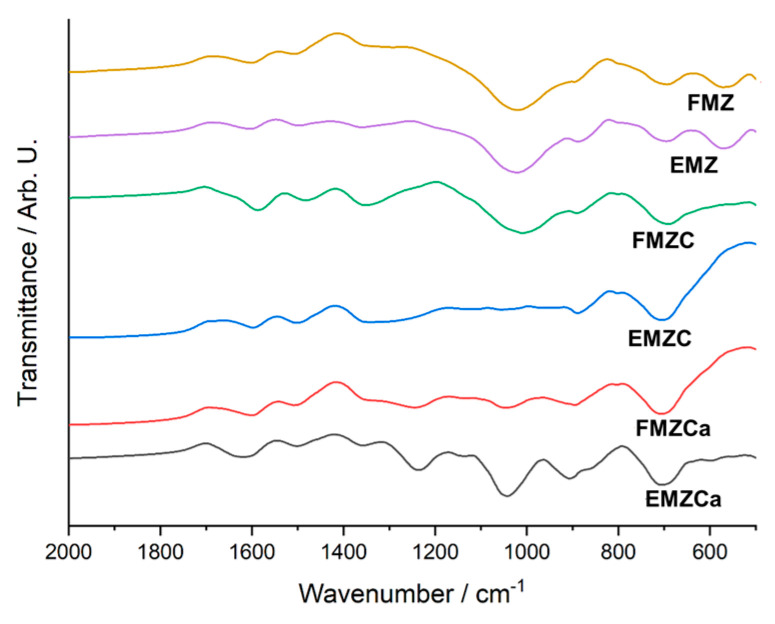
FT-IR spectrum of FMZ, EMZ, FMZCa, EMZCa, FMZC, and EMZC after adsorption.

The FTIR spectra of the prepared adsorbents reveal significant changes following chromium adsorption (Figure 14).

In the FMZ spectra, notable shifts are observed in the adsorption peaks at 830 cm^−1^, assigned to AlO_4_ coordination, and around 1066 cm^−1^, corresponding to the Si-O asymmetric stretch. All these shifts are accompanied by alterations in peak intensities. For EMZ, vibrational band shifts are detected at approximately 880 cm^−1^ and 1418 cm^−1^ (C-O stretch), 1066 cm^−1^ (Si-O asymmetric stretch), 1637 cm^−1^ (C=O stretching), 1643 cm^−1^ (N-H stretching), and 1077 cm^−1^ (C-N stretching). Such spectral changes could be attributed to the chemical interactions between chromium ions and the specific functional groups present in FMZ and EMZ. Moreover, both k-carrageenan-based nanocomposites (FMZCa and EMZCa) show significant shifts in the absorption bands within the sulfate ester region, particularly at 1454, 1394, 1266, and 848 cm^−1^. In contrast, the FTIR spectra of chitosan-based nanocomposite display primarily shift at approximately 1568, 1413, 1018, and 870 cm^−1^, which are associated with the ammonium groups in the chitosan structure. These observed spectral alterations further emphasize the interactions between chromium ions and the functional groups within the biopolymer-based nanocomposites [30].

#### 3.11.2. SEM-EDX Analysis

**Figure 15:**
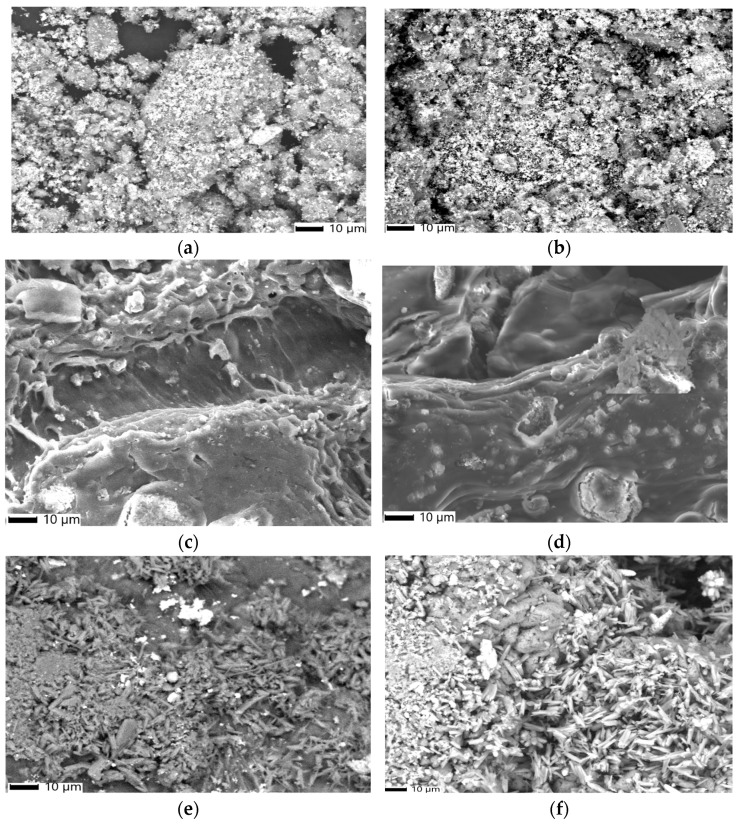
SEM images of FZM (**a**), EMZ (**b**), FMZCa (**c**), EMZCa (**d**), FMZC (**e**), and EMZC (**f**) after adsorption.

**Figure 16:**
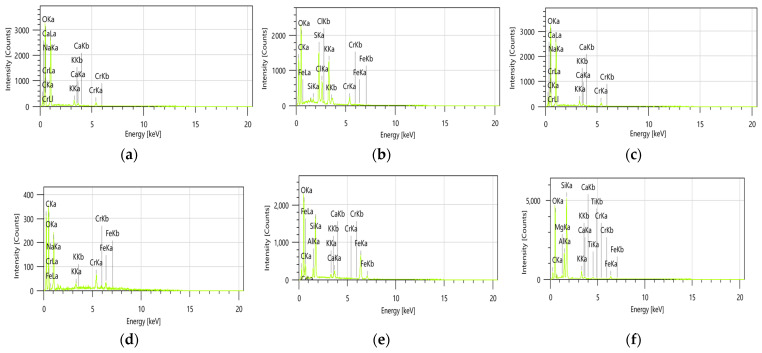
EDX spectrum of EMZ (**a**), FMZ (**b**), EMZCa (**c**), FMZCa (**d**), EMZC (**e**), and FMZC (**f**) after adsorption.

Morphological changes in all prepared adsorbent types (FMZ, EMZ, EMZCa, FMZC, and EMZC) after chromium adsorption were evaluated through SEM-EDX analysis (Figure 15 and Figure 16). This assessment aimed to support the findings from FT-IR spectroscopy by examining variations in particle size, shape, pore dimensions, and particle distribution.

The SEM micrographs of FMZ and EMZ following chromium adsorption (Figure 15) illustrate the presence of numerous irregularly shaped particles attributed to the pollutant. Additionally, the SEM images indicate a discernible reduction in the porosity of these adsorbents after chromium uptake. For the biopolymer-based nanocomposites (FMZCa, EMZCa, FMZC, and EMZC), the micrographs reveal clusters of irregularly shaped particles that are unevenly distributed within the polymeric matrix, accompanied by observable modifications in the matrix morphology. These findings suggest significant alterations resulting from the adsorption process.

#### 3.11.3. EDX Analysis

Elemental analysis (EDX) of all prepared adsorbents following chromium adsorption was conducted (Figure 16). The EDX results indicated a distinct peak corresponding to the heavy metal, thereby confirming the successful adsorption of chromium [69,70].

**Figure 17:**
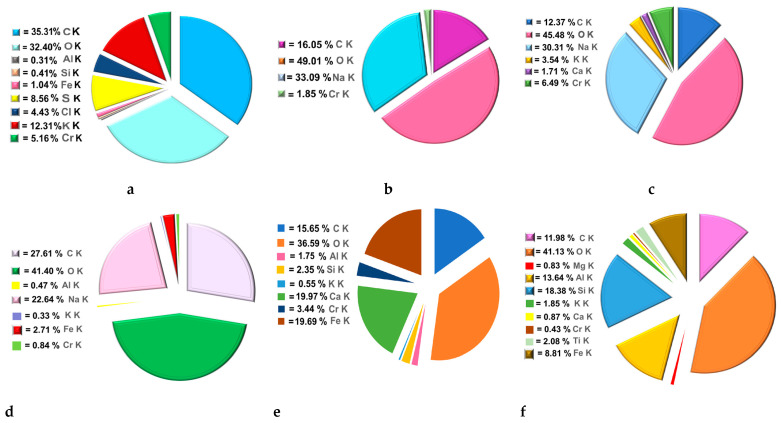
Elemental composition (%) determined via EDX analysis for EMZCa (**a**); FMZCa (**b**); EMZC (**c**); FMZC (**d**); EMZ (**e**), and FMZ (**f**).

The elemental composition (%) determined via EDX analysis (Figure 17) reveals distinct variations in the chromium (Cr) retention across the surfaces of different types of adsorbent prepared.

#### 3.11.4. Thermal Analysis

**Figure 18:**
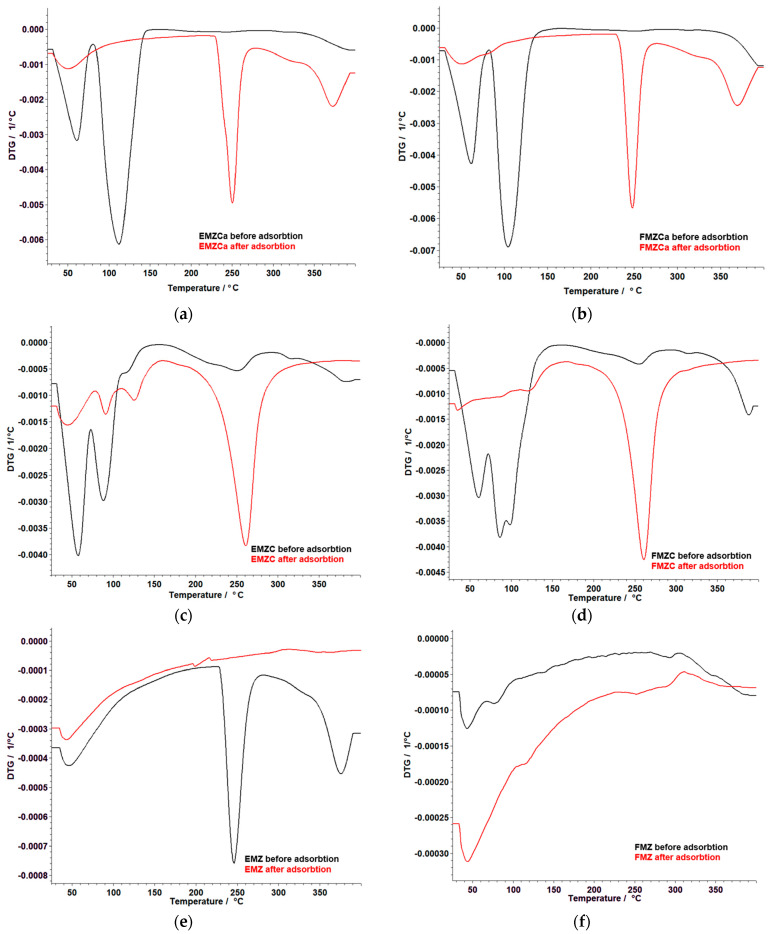
Comparative DTG curves of all prepared adsorbents before/after adsorption: EMZCa (**a**); FMZCa (**b**), EMZC (**c**), FMZC (**d**), EMZ (**e**), and FMZ (**f**).

Thermal analysis of all types of adsorbents prepared after chromium adsorption was performed to evaluate the changes in thermal stability after chromium adsorption. The results are illustrated in Table 7 and Figure 18.

Thermal analysis of prepared adsorbents after chromium retention indicates distinct thermal decomposition behaviors depending on the biopolymer matrices and composition. Adsorbents FMZC and EMZ exhibit a two-stage thermal degradation process, wherein the initial phase is characterized by moisture loss, followed by the degradation of the polymer membrane. In contrast, adsorbents EMZCa, FMZCa, EMZC, and EMZ are observed to undergo a more intricate three-stage thermal decomposition. For k-carrageenan-based nanocomposites (EMZCa and FMZCa), the first stage involves moisture loss, followed by water removal from the membrane matrix and degradation of the polymer. This process initiates at temperatures above 200 °C and continues to progress at temperatures exceeding 300 °C. The chitosan-based nanocomposite, EMZC, also undergoes a three-stage decomposition process. The initial phase involves moisture loss, followed by the degradation of the polymer matrix, and concludes with the decomposition of organic compounds present in the eggshell particulates. Conversely, the thermal analysis of FMZC reveals a two-stage mechanism assigned to the loss of moisture and the subsequent degradation of the polymer membrane. The thermal behavior of nanocomposite EMZ is characterized by two stages as well, with the initial phase involving moisture loss, followed by the thermal degradation of organic components derived from the eggshell matrix. Finally, FMZ undergoes a three-stage thermal decomposition process, which includes the loss of water, followed by the elimination of crystallization water and the decarbonation of zeolite components [29,71].

The thermal analysis results demonstrate that carrageenan-based nanocomposites exhibit enhanced thermal stability in the formulation of k-carrageenan-based nanocomposite EMZCa compared to FMZCa. In a parallel observation, chitosan-based nanocomposites in formulation EMZC show superior thermal stability to FMZC, although EMZC exhibits a higher total mass loss of 0.75%. This discrepancy can be attributed to the higher proportion of chromium ions retained on this specific adsorbent. These conclusions corroborate with data derived from the corresponding adsorption studies.

### 3.12. Adsorption Mechanism

Various studies reported three predominant mechanisms (ion exchange, electrostatic attraction, and reduction) for chromium removal from aqueous media [1,9].

In this study, we identified an optimal pH of 4.5, where the coexistence of dichromate (Cr^2^O_7_^2−^) and hydrogen chromate (HCrO_4_^−^) ions occurs. In this acidic environment, the complex structures of FMZ and EMZ promote the immobilization of chromium through the electrostatic attraction of chromate anions to the divalent and trivalent cations, specifically Ca^2+^, Mg^2+^, and Fe^3+^, present in these adsorbents. Additionally, chromium ions can penetrate the pores of zeolite, where steric effects, such as van der Waals interactions, facilitate their confinement.

The protonated NH groups in eggshell particles, stemming from the protein components of the eggshell membrane, facilitate the attraction of chromium ions and forming surface complexes via chemical bonding on EMZ adsorbent [4].

Concurrently, the reduction of HCrO_4_^−^ ions by Fe(II) ions sourced from magnetite and protons occurs alongside the exchange of chromium ions from the aqueous solution with Na^+^, K^+^, and Mg^2+^ from EMZ and FMZ [13]. These reflect chemisorption activity, supported by prior spectroscopic, kinetic, and thermodynamic studies (vide supra). In addition, zeolite can trap Cr^3+^ ions, promoting immobilization into the FMZ and EMZ tertiary matrix [22,72]. Concomitantly, in the case of chitosan-based nanocomposites, specifically FMZC and EMZC, the amino (-NH_2_) and hydroxyl (-OH) groups in the polymer matrix undergo complete protonation to form –NH_3_^+^ and –OH_2_^+^ species. These positively charged groups exhibit a strong affinity for the chromium ions from aqueous solution, resulting in surface-bound complexes through chemical bonding. Furthermore, Cr(VI) ions are reduced in the presence of Fe(II) and protons, which facilitates the adsorption of the resulting Cr(III) ions within the chitosan matrix. This process ultimately results in covalent bond formation, specifically –H_2_N–Cr(III), characterized by a coordinate bond involving the non-bonding electrons of nitrogen.

**Figure 19:**
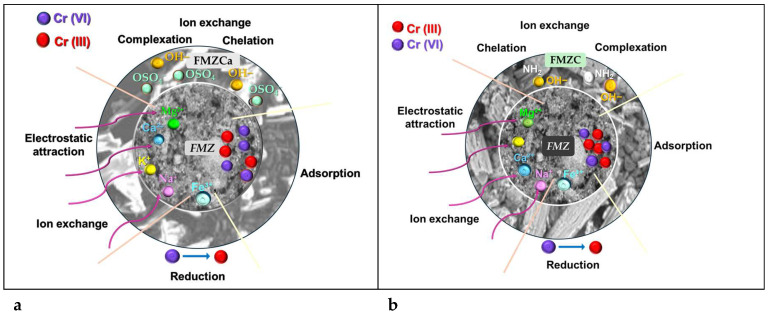

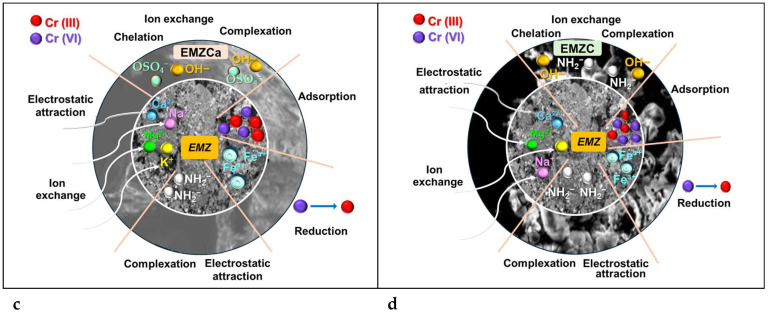
Schematic representation of the adsorption mechanism of prepared adsorbents: FMZ/FMCa (**a**), FMZ/FMZC (**b**) EMZ/EMZCa (**c**), and EMZ/EMZC (**d**).

In the context of k-carrageenan-based nanocomposites, specifically FMZCa and EMZCa, the mechanism for the removal of chromium (VI) ions involves multiple simultaneous processes: ion exchange, electrostatic attraction, reduction, and chelation. At the optimal pH established for this study, hydroxyl ions (OH−) present on the adsorbent surface are readily substituted by HCrO_4_^−^ ions. In an acidic environment, the protonation of hydroxyl and sulfate groups within the biopolymer enhances the availability of adsorption sites. This modification significantly strengthens the electrostatic interactions with chromium (VI) ions. The abundance of protons (H^+^) facilitates the attraction of hydroxyl groups from k-carrageenan, while at the same time, HCrO_4_^−^ ions are repelled from the –OH^−^ groups due to the governing electrostatic forces. Furthermore, under the influence of protons and Fe(II) ions, hydrogen chromate (HCrO_4_^−^) is reduced to Cr^3+^ ions, which are subsequently attracted to the sulfate ions (–SO_4_^−^) and adsorbed on the polymer matrix. Additionally, the ionic OSO_3_^−^ groups facilitate the formation of metal-ligand complexes with chromium (VI) ions [30,73,74,75]. A schematic representation of chromium adsorption on all prepared adsorbents is presented in Figure 19.

### 3.13. Comparison of Chromium Removal Efficiency Among Other Adsorbents

A comparative evaluation of the adsorption performance for all prepared adsorbent types, as well as those reported in the relevant literature concerning chromium removal, is presented in Table 8.

The results indicate that all three categories of adsorbents, namely the nanocomposites (EMZ and FMZ), the k-carrageenan-based nanocomposites (EMZCa and FMZCa), and the chitosan-based nanocomposites (EMZC and FMZC) exhibit significantly higher adsorption efficiencies than any recognized materials. This increase can be attributed to their greater surface areas compared to their constituent components, such as magnetite, zeolite, and eggshell or fly ash, in addition to the enhancements made to the biopolymeric matrices following the modification of the adsorbent surface efficiency than any other known materials. This could be attributed to the higher surface area compared to their components (eggshell or zeolite), following adsorbent surface modification [5,84,85].

### 3.14. Desorption Studies

**Figure 20:**
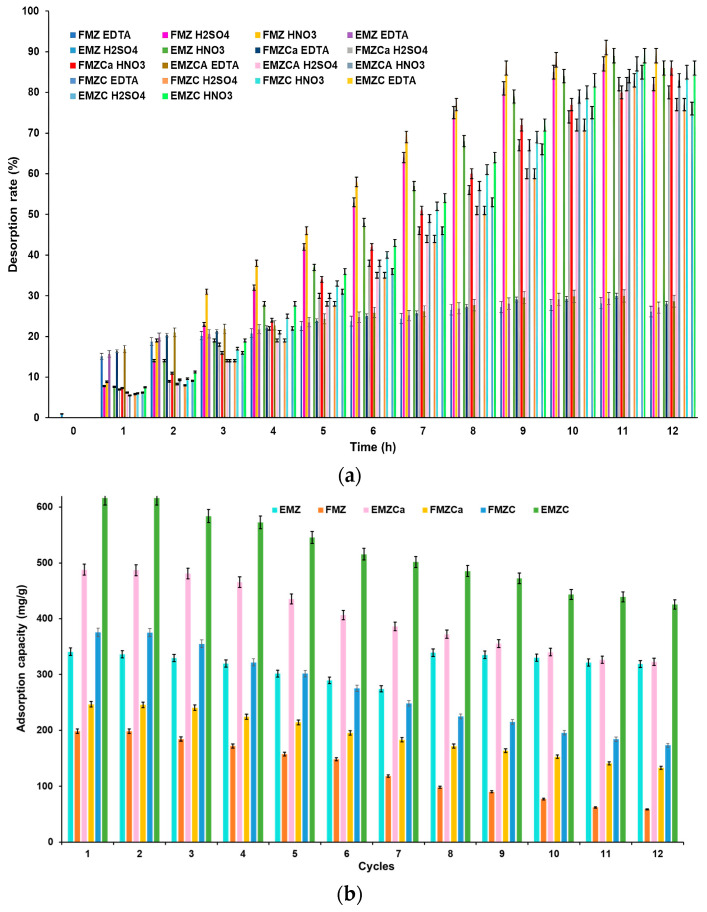
(**a**) Relationship between desorption rate and time. (**b**) Reusability of all prepared adsorbents.

Cycling stability is pivotal to achieving high-performance and cost-effective adsorbents [86]. The regeneration efficiency is significantly influenced by the desorption kinetics of the adsorbed pollutants [40]. In this study, the desorption of chromium ions was examined under acidic conditions, specifically utilizing nitric acid, sulfuric acid, and the chelating agent ethylenediaminetetraacetic acid (EDTA). The results (Figure 20) indicate that in an acidic environment, the desorption rate increases markedly within the 0 to 11-h timeframe, reaching a maximum, after which a slight decline in desorption yield is observed over time.

A comprehensive desorption study was conducted using three distinct desorption agents (Figure 20a). Applying 0.1 N EDTA resulted in the release of approximately 40% of the chromium adsorbed onto chitosan-based nanocomposites, with specific releases of 40.02% for EMZC and 39.85% for FMZC. In comparison, the release rates for FMZ and EMZ were lower, at approximately 28.15% and 29.5%, respectively. The desorption outcomes for k-carrageenan-based nanocomposites approached 30%, with recorded values of 29.98% for FMZCa and 30.01% for EMZCa, predominantly as Cr(III) ions. These findings can be attributed to the formation of polydentate coordination bonds with Cr(III) ions, thereby enhancing the effective recovery through interactions with the functional groups from the biopolymeric matrices. Furthermore, EDTA is a well-established chelating agent known for its effectiveness in regenerating chitosan [87].

The utilization of 0.1 N mineral acids demonstrated significant desorption rates, with between 89% and 91% of the adsorbed chromium released into solution as Cr(III) ions, specifically recording 91% for EMZ and 89% for FMZ when using HNO_3_. The desorption rates were higher for the nanocomposites (91% for EMZ and 89% for FMZ), followed by chitosan-based nanocomposites (with rates of 85% for EMZC and 83% for FMZ), and k-carrageenan-based composites (with 82% for EMZCa and 80% for FMZCa), during the application of 0.1 M HNO_3_.

Similar results were obtained with 0.1 M H_2_SO_4_, where over 80% of the heavy metal was desorbed from all adsorbents, with the highest desorption rate of 89% observed for EMZC. This outcome for chitosan-based nanocomposites suggests that HCrO_4_^−^ ions adsorbed by the prepared adsorbents were reduced to Cr(III) during desorption by mineral acids, thereby releasing Cr(III) ions into the solution. This phenomenon can be explained by repulsion from the protonated functional groups within the biopolymer matrix [88].

Conversely, the outcomes for other adsorbent types evaluated in this study may be attributed to ion exchange interactions, which could be more prevalent than chemical sorption [89].

To that end, thirteen chromium adsorption and desorption cycles were conducted on both adsorbents to investigate their reusability potential.

## 4. Discussion

We have successfully prepared two novel composite nano adsorbents: one composed of fly ash, zeolite, and magnetite (FMZ) and the other consisting of eggshell, zeolite, and magnetite (EMZ). Building upon these foundational composite nanoadsorbents, we developed two k-carrageenan-based composites (FMZCa and EMZCa) and two chitosan-based composites (FMZC and EMZC). The preparation of these biopolymer-based composites involves meticulously loading FMZ and EMZ into k-carrageenan and chitosan matrices, respectively. The XRD spectrum demonstrated that FMZ and EMZ composites exploit the high surface area and porosity of zeolite and the magnetic properties of magnetite, enhancing the efficiency of pollutant removal compared to the performance of their starting components. The subsequent k-carrageenan and chitosan modifications further augment the adsorption capacities and thermal stability. The VSM study indicated a predominantly ferromagnetic profile for the samples, with the notable exceptions of FMZCa and EMZC, which displayed a hysteretic profile exhibiting paramagnetic behavior near zero fields.

Thermal analysis revealed that although FMZ is more stable than EMZ, after loading into the polymer matrix, EMZ is more thermally stable than FMZ, regardless of the biopolymer type used.

These innovative adsorbents capitalize on the synergistic properties of their constituents: fly ash and eggshells serve as sustainable bases, promoting waste valorization while maximizing the capabilities of zeolite and magnetite for pollutant removal. Moreover, the subsequent k-carrageenan and chitosan modifications further enhance their adsorption capabilities and mechanical integrity, yielding robust materials suitable for addressing diverse environmental challenges.

The adsorption behavior of all new adsorbent types follows the Freundlich isotherm and pseudo-second-order models. The adsorption efficiency of each adsorbent type exceeds 84% of Cr(VI), (89.76 for EMZ, and 84.83% for FMZ), more than 94% of Cr(VI) for each k-carrageenan-based composite (97.67% for EMZCa and 94.87% for FMZCa) and over 96% for each chitosan-based composite (99.64% for EMZC and 96.36% for FMZC).

According to this study’s results, EMZC (chitosan-based composite) and EMZCa (k-carrageenan-based composite) demonstrate higher adsorption capacity for chromium (487.82 mg/g for EMZCa and 616.19 mg/g for EMZC) compared to other composites prepared in this study due to the synergistic effects of polymer matri-ces and their components. Thus, both EMZC and EMZCa provide a higher density of functional groups (e.g., -NH_3_^+^, -OH_2_^+^ in EMZC; -SO_4_^−^, -OH in EMZCa) compared to unmodified composites like EMZ or FMZ, offering more adsorption sites for chromium. Furthermore, the combination of protonated biopolymers and their components (magnetite, zeolite, eggshell) create a cooperative system where ad-sorption is enhanced through multiple mechanisms, including reduction, ion ex-change, and chemical bonding. In addition, the polymer matrices improve the dispersion of inorganic components, exposing more active sites for chromium binding and increasing the overall surface area of the composite. Conversely, the higher adsorption capacities of EMZC and EMZCa compared to FMZC and FMZCa can be attributed to the structural and compositional differences between eggshell-based (EMZ) and fly ash-based (FMZ) composites. Eggshells contain a high concentration of calcium carbonate (CaCO_3_) and organic proteinaceous ma-terials, which enhance adsorption through multiple mechanisms. In EMZC and EMZCa, the calcium ions (Ca^2+^) from the eggshell contribute significantly to the electrostatic attraction of chromate anions (Cr_2_O_7_^2−^, HCrO_4_^−^) and facilitate ion ex-change processes. Additionally, the proteinaceous components in eggshells, such as the amine-rich eggshell membrane, further support chemical bonding and sur-face complexation with chromium ions, providing an additional pathway for ad-sorption.

In contrast, FMZ composites rely on fly ash, primarily composed of silica and alumina, which are less interactive in chromium adsorption due to the lack of or-ganic functional groups and lower ion exchange potential. Furthermore, EMZ composites benefit from their more heterogeneous and porous structure, which increases the surface area and accessibility of active sites for adsorption. When combined with chitosan or k-carrageenan matrix, the enhanced functional group density and chemical reactivity of EMZC and EMZCa allow for superior binding of chromium ions compared to FMZC and FMZCa. These factors collectively ex-plain the greater adsorption capacities of EMZ-based composites.

In comparison with the literature on similar materials to their starting components, the three types of new materials (EMZ, FMZ, EMZCa, FMZCa, EMZC, and FMZC) demonstrate higher absorption capacity attributed to the higher surface and the microporous structure resulting from the used experimental conditions exhibit higher adsorption efficiency [21,22,29,44,52,70].

The potential applications of these composites and biocomposites span water purification and pollutant sequestration, thereby providing effective solutions to pressing global environmental issues through cutting-edge nanotechnology.

The materials derived from this research demonstrate stability and ease of reuse, further underscoring their applicability in wastewater remediation within a sustainable economy. Collectively, these novel adsorbent types present significant advancements in the environmental engineering field.

## 5. Conclusions

This study investigates the efficacy of chromium removal from aqueous solutions utilizing three novel adsorbent types: (i) Nanocomposites EMZ and FMZ, developed from eggshell waste, which is integrated with zeolite and magnetite, and from fly ash, which is combined with zeolite and magnetite, respectively; (ii) k-carrageenan-based composites EMZCa and FMZCa, prepared by incorporating the aforementioned nanocomposites into a k-carrageenan matrix; (iii) Chitosan-based composites, formed by embedding the newly created composites into a chitosan matrix.

The functionalization of EMZ involved zeolite and magnetite particles, which were loaded within the pores of the eggshell and validated through SEM, XRD, and FTIR analysis. For FMZ, the approach involved loading fly ash and magnetite particles into zeolite pores. The polymer-based composites underwent similar functionalization, ensuring that each nanocomposite was effectively loaded into the polymer matrix. XRD analysis revealed a notable reduction in crystallinity for both biopolymer composites, correlating with an increase in specific surface area. SEM and BET analysis corroborated these structural modifications across all newly developed adsorbents. Consequently, EMZC exhibited superior adsorption performance (99.64%, 616.19 mg/g) compared to EMZCa (97.67%, 246.53 mg/g) and EMZ (89.76%, 340.84 mg/g).

Similarly, FMZC (97.67%, 375.45 mg/g) outperformed FMZCa (94.87%, 246.53 mg/g) and FMZ (84.83%, 198.47 mg/g), achieving a maximum adsorption capacity of 96.365 mg/g. Optimal adsorption conditions were identified at 25 °C, pH 4.5, and had a contact time of 120 min. The adsorption process was characterized by isotherm, thermodynamic, and kinetic modeling, suggesting a physisorption mechanism interlaced with a chemical adsorption component, which fitted well with a second-order kinetics model. Further analysis using SEM, FT-IR, and DTG post-metal adsorption indicated that chromium uptake occurred through the formation of chemical bonds.

Regeneration experiments demonstrated that chromium could be efficiently desorbed from the adsorbents’ surfaces using mineral acids, with maximum desorption rates of 91% for EMF and 89% for FMZ, which were followed by 85% and 83% for the chitosan-based composites EMZC and FMZC and 82% and 80% for the k-carrageenan-based composites EMZCa and FMZCa.

Overall, the findings present a thorough characterization of the newly prepared composites and biopolymer-based composites, which exhibit attributes of high performance, selectivity, recyclability, cost-efficiency, and eco-friendliness, thereby rendering them suitable for applications in wastewater remediation. This study proposes an innovative ecological approach leveraging waste materials for effective chromium immobilization from aqueous environments.

Future research endeavors should focus on the following areas: (a) The evaluation of the performance of these newly developed adsorbents under real-world conditions, encompassing their interactions with other heavy metals and organic pollutants; (b) The investigation of their long-term stability and efficacy in dynamic systems, particularly under continuous flow conditions and fluctuating pH levels over extended durations; (c) The economic feasibility of scaling up production processes.

## 6. Patents

A patent application was submitted to the Romanian State Office for Inventions and Trademarks and recorded as A/0001/2024.

## Figures and Tables

**Table 1:** BET-determined parameters of the newly prepared nanocomposites and their components *.

Sample	Surface Area (m^2^/g)	Average Pore Size Diameter (nm)	Total Pore Volume (cm^3^/g)
chitosan	42.77	2.08	9.21 × 10^−3^
k-carrageenan	129.86	3.79	11.35 × 10^−3^
eggshell	1.328	8.423	2.05 × 10^−3^
magnetite	18.02	3.81	44.78 × 10^−3^
fly ash	4.959	8.987	20.19 × 10^−3^
zeolite	12.126	15.615	38.13 × 10^−3^
FMZ	31.42	7.89	10.16 × 10^−3^
EMZ	34.79	5.57	10.03 × 10^−3^
FMZCa	142.77	11.02	47.49 × 10^−3^
EMZCa	145.34	13.88	50.77 × 10^−3^
FMZC	196.12	12.83	49.23 × 10^−3^
EMZC	201.83	14.89	51.34 × 10^−3^

* standard deviation (SD) = 2%.

**Table 2:** VSM results for all prepared adsorbents.

Samples	Coercivity (Oe)	Remanent Magnetization (emu/g)	Saturation Magnetization (emu/g)
FMZCa	100.78	0.23	2.2317
FMZC	103.30	1.19	10.593
FMZ	101.82	2.77	21.545
EMZCa	101.32	0.95	8.2122
EMZC	102.43	0.08	0.79719
EMZ	101.76	3.94	29.775

**Table 3:** Thermal analysis results for all types of prepared adsorbents.

Sample	Process	TG		DTG_max_/°C	Δm/%	Total Mass Loss %
		T_onset_/°C	T_final_/°C			
EMZCa	I	37	80	60	8.89	31.22
	II	85	144	115	20.96	
FMZCa	I	34	81	61	12.23	34.73
	II	82	140	139	19.76	
EMZC	I	35	72	58	11.16	28.67
	II	71	132	89	8.72	
	III	287	397	-	4.71	
FMZC	I	33	69	62	8.48	30.25
	II	71	124	90	14.07	
	III	290	396	390	4.23	
EMZ	I	32	80	54	1.97	8.68
	II	223	274	248	1.82	
	III	349	400	380	1.72	
FMZ	I	34	91	78	0.6	1.72
	II	96	188	-	0.39	

**Table 4:** The Langmuir and Freundlich model parameters for chromium adsorption on each prepared adsorbent.

Adsorbent	Langmuir Model					Freundlich Model			
	Q_e,exp_	Q_m_	k	R_L_	R^2^	K_F_	*n*	R^2^	E_a_ (kJ/mol)
EMZ	340.84	339.83	0.189	0.602	0.9964	2.241	1.393	0.9981	32.61
FMZ	198.47	197.52	0.185	0.683	0.9882	2.252	1.401	0.9956	32.35
EMZCa	487.82	487.17	0.191	0.486	0.9933	2.824	1.551	0.9951	32.72
FMZCa	246.53	245.72	0.197	0.506	0.9849	2.977	1.473	0.9897	32.79
EMZC	375.45	374.22	0.186	0.489	0.9957	3.365	1.527	0.9965	32.19
FMZC	616.19	615.89	0.195	0.522	0.9938	3.016	1.673	0.9962	32.22

**Table 5:** Thermodynamic parameters for chromium adsorption on all prepared adsorbent types *.

Adsorbent	ΔG^0^ (kJ/mol)			ΔH^0^ (kJ/mol)	ΔS^0^ (J/(mol K))
	T(K)				
	295.15	303.15	313.15		
EMZ	−17.11	−18.32	−19.83	27.48	151.09
FMZ	−14.33	−15.30	−16.51	21.41	121.11
EMZCa	−24.90	−26.75	−29.06	43.30	231.08
FMZCa	−21.54	−23.12	−25.08	36.49	196.64
EMZC	−32.65	−35.13	38.22	58.53	308.96
FMZC	−20.26	−20.60	−21.03	47.56	143.04

* standard deviation (SD) = 2%.

**Table 6:** Kinetic parameters for chromium adsorption on EMZ, FMZ, EMZCa, FMZCa, EMZC, and FMZC adsorbents *.

Adsorbent Material	Q_e_^exp^ (mg/g)	Pseudo-First Order			Pseudo-Second Order			Intraparticle Diffusion		
		q_e_^calc^	K_1_	R^2^	Q_e_^calc^	K_2_	R^2^	K_i_	C	R^2^
EMZ	340.84	341.04	0.197	0.989	341.38	8.462	0.987	3.485	15.938	0.975
FMZ	198.47	198.86	0.216	0.975	199.21	2.017	0.976	1.770	4.709	0.963
EMZCa	487.82	489.32	0.194	0.991	489.44	8.058	0.988	5.545	32.443	0.971
FMZCa	246.53	246.96	0.208	0.986	247.18	2.267	0.985	2.598	16.529	0.961
FMZC	375.45	376.23	0.192	0.977	376.41	1.053	0.975	4.316	20.742	0.976
EMZC	616.19	619.78	0.211	0.987	617.36	6.568	0.986	7.255	33.315	0.986

* standard deviation (SD) = 2%.

**Table 7:** Thermal analysis results for all types of prepared adsorbents after chromium retention.

Sample	Process	TG		DTG_max_/°C	Δm/%	Total Mass Loss %
		T_onset_/°C	T_final_/°C			
EMZCa	I	35	93	51	5.48	33.06
	II	213	292	250	10.95	
	III	331	400	380	10.09	
FMZCa	I	36	97	-	5.92	33.82
	II	220	279	249	10.03	
	III	335	400	372	10.57	
EMZC	I	33	78	54	6.56	35.2
	II	79	141	96	6.18	
	III	200	320	264	17.29	
FMZC	I	36	114	63	9.04	34.62
	II	223	300	261	15.10	
EMZ	I	35	66	-	1.03	33.89
	II	184	257	210	0.49	
FMZ	I	39	114	-	1.91	4.22
	II	173	258	-	0.688	
	III	297	370	-	0.40	

**Table 8:** Comparison between chromium removal efficiency of the new types of adsorbents and those reported in the literature (selected studies) for materials similar to their components.

Adsorbent Type	Removal Performance	Chromium Initial Concentration	Ref.
eggshell	285.71 mg/g	300 mg/L	[58]
chitosan flakes	138.45 mg/g	500 mg/L	[75]
montmorillonite/carrageenan (MMT/CG)-composite hydrogel	106 mg/g	80 mg/L	[76]
fly ash	83.9%	20 mg/L	[14]
Fe_3_O_4_ nanoparticles	80.4%	1000 mg/L	[77]
MWCNT/Fe_3_O_4_	89.1%	1000 mg/L	[78]
natural zeolite (ZN-NaOH)	44.44%	0.6 mg/L	[79]
synthetic zeolite (ZS-NaOH)	32.22%	0.6 mg/L	[79]
clinoptilolite treated with NaCl	4.5 mg/g	10 mg/L	[80]
clinoptilolite	2.2 mg/g	10 mg/L	[80]
ZnO-chitosan nano-biocomposite	96.5% (65 mg/g)	20 mg/L	[81]
FeS/CTS	99.18%	50 mg/L	[82]
CS-AL nanocomposites with glutaraldehyde	79.91%	100 mg/L	[83]
FMZ	84.83% (198.47 mg/g)	28.5 mg/L	This study
EMZ	89.76% (349.84 mg/g)	28.5 mg/L	This study
FMZCa	94.87% (246.53 mg/g)	28.5 mg/L	This study
EMZCa	96.36% (487.82 mg/g)	28.5 mg/L	This study
FMZC	97.67% (375.45 mg/g)	28.5 mg/L	This study
EMZC	99.64% (616.19 mg/g)	28.5 mg/L	This study

## Data Availability

The datasets and relevant material used and/or analyzed during the current study are available from the corresponding author upon reasonable request.

## Abbreviations

WHO	World Health Organization
BET	Brunauer–Emmett–Teller method
XRD	X-ray diffraction analysis
FTIR	Fourier-transform infrared spectroscopy
VSM	vibrating sample magnetometer
SEM	scanning electron microscopy
Ess	eggshell
FMZ	tertiary composite composed of fly ash, magnetite, and zeolite
EMZ	tertiary composite containing eggshell, magnetite, and zeolite
WPPF	whole powder pattern fitting method
BJH	Barrett-Joyner-Halenda
FEG	field emission gun
FMZC	biopolymer-nanocomposite material based on fly ash, magnetite, and zeolite, loaded in chitosan matrix
EMZC	biopolymer-nanocomposite material based on eggshell, magnetite, and zeolite, loaded in chitosan
FMZCa	biopolymer-nanocomposite material based on fly ash, magnetite, and zeolite, loaded in k-carrageenan
EMZCa	biopolymer-nanocomposite material based on eggshell, magnetite, and zeolite, loaded in k-carrageenan
